# An Estuarine Cyanophage S-CREM1 Encodes Three Distinct Antitoxin Genes and a Large Number of Non-Coding RNA Genes

**DOI:** 10.3390/v15020380

**Published:** 2023-01-28

**Authors:** Hongrui Zheng, Yuanfang Liu, Ruiyu Zhou, Jihua Liu, Yongle Xu, Feng Chen

**Affiliations:** 1 Institute of Marine Science and Technology, Shandong University, Qingdao 266000, China; 2 Institute of Marine and Environmental Technology, University of Maryland Center for Environmental Science, Baltimore, MD 21202, USA

**Keywords:** cyanophage, new phage genus, antitoxin genes, non-coding RNA genes

## Abstract

Cyanophages play important roles in regulating the population dynamics, community structure, metabolism, and evolution of cyanobacteria in aquatic ecosystems. Here, we report the genomic analysis of an estuarine cyanophage, S-CREM1, which represents a new genus of T4-like cyanomyovirus and exhibits new genetic characteristics. S-CREM1 is a lytic phage which infects estuarine *Synechococcus* sp. CB0101. In contrast to many cyanomyoviruses that usually have a broad host range, S-CREM1 only infected the original host strain. In addition to cyanophage-featured auxiliary metabolic genes (AMGs), S-CREM1 also contains unique AMGs, including three antitoxin genes, a MoxR family ATPase gene, and a pyrimidine dimer DNA glycosylase gene. The finding of three antitoxin genes in S-CREM1 implies a possible phage control of host cells during infection. One small RNA (sRNA) gene and three *cis*-regulatory RNA genes in the S-CREM1 genome suggest potential molecular regulations of host metabolism by the phage. In addition, S-CREM1 contains a large number of tRNA genes which may reflect a genomic adaption to the nutrient-rich environment. Our study suggests that we are still far from understanding the viral diversity in nature, and the complicated virus–host interactions remain to be discovered. The isolation and characterization of S-CREM1 further our understanding of the gene diversity of cyanophages and phage–host interactions in the estuarine environment.

## 1. Introduction

Picocyanobacteria of the genera *Synechococcus* and *Prochlorococcus* are important primary producers in a wide range of marine environments [1,2]. In estuaries, picocyanobacteria contribute up to 56% of the primary production [3]. Cyanophages, viruses infecting cyanobacteria, are prevalent in marine ecosystems, lysing up to 40% of *Synechococcus* cells every day, playing a key role in regulating the population dynamics of the cyanobacteria [4,5]. To date, all cyanophages isolated from marine ecosystems belong to the class *Caudovirales*, including *Myoviridae*, *Podoviridae*, *Siphoviridae*, and the families *Ackermannviridae*, and *Herelleviridae* [6]. Cyanomyoviruses are the most frequently isolated cyanophages in marine ecosystems, with T4-like cyanophages as the most important group [7,8]. T4-like cyanophages usually contain a set of core genes including virion formation and DNA replication genes [7]. In addition, T4-like cyanophages generally encode various auxiliary metabolic genes (AMGs), and they can affect host photosynthesis, carbon metabolism, nutrient acquisition, stress tolerance, and nucleic acid synthesis during infection [9,10,11,12,13]. AMGs in T4-like cyanophages indicate the characteristics of phage–host interactions, and the gain or loss of AMGs in the cyanophage genomes is the adaptation to environmental selection pressures [14]. tRNA genes are widely present in the T4-like cyanophage genomes, with the number varying greatly [7]. tRNAs may play roles in improving the phage cross-infectivity of different hosts and in replacing host tRNAs to maintain translation [15,16]. Furthermore, other non-coding RNA genes, including CFrI, PhotoRC-II, *wcaG*, and the glutamine riboswitch, are also found in T4-like cyanophage genomes, which are predicted to influence the host’s metabolism [8,17,18].

Estuaries connect marine and freshwater systems. Environmental gradients in estuaries are dynamic and pose great selective pressures on microorganisms [6,19]. Metagenomic studies revealed significant differences between estuarine phages and open ocean phages [20]. Estuarine cyanophages have developed unique genomic characteristics to cope with the highly dynamic environment [21,22]. The estuarine *Synechococcus* phage S-CBWM1 has a unique set of AMGs, structural, and DNA replication genes, and it possesses the largest number of tRNA genes that have ever been found in cyanophage isolates [22]. Notably, phages with more than 20 tRNAs are mainly isolated from eutrophic environments [22,23,24]. In addition, cyanophages isolated from eutrophic waters may carry fewer and more unique AMGs [25,26]. The gain and loss of unique AMGs in cyanophage genomes can be attributed to the fluctuating selection pressures in the estuarine environment [14], resulting in different host host–phage interactions compared to those in the marine environment [6]. However, only a few estuarine cyanophages have been isolated and studied, which limits our understanding of estuarine cyanophages’ evolution and ecological roles. The isolation, genomic and physiological characterizations of new viruses are still of great significance for further exploring genetic diversity, understanding virus host–phage interactions, and elucidating their ecological roles in estuarine ecosystems [27,28].

Here, we characterized a newly isolated estuarine *Synechococcus* phage, S-CREM1. S-CREM1 represents a new genus of the T4-like cyanomyoviruses, and it carries a unique set of AMGs and various non-coding RNA genes. The isolation and characterization of S-CREM1 provide new insights into phage–host interactions in the estuarine environment.

## 2. Materials and Methods

### 2.1. Host Incubation and Cyanophage Isolation

Host strain *Synechococcus* sp. CB0101 isolated from Chesapeake Bay is a model strain of picocyanobacteria in the estuarine environment and belongs to *Synechococcus* subcluster 5.2. *Synechococcus* sp. CB0101 was grown in SN medium with 15‰ salinity (SN15), at 22 ℃, under a constant light intensity of 20 µmol photons m^−2^ s^−1^ in an illumination incubator [29,30]. S-CREM1 was isolated from the surface seawater of the Changjiang River Estuary (30.8° N, 122.6° E) using *Synechococcus* sp. CB0101 as the host [21]. The phage-containing seawater sample was collected from the surface of the Changjiang River Estuary and filtered through a 0.22 µm pore-size polycarbonate membrane (Millipore, Bedford, MA, USA) to remove microbial cells. The filtered sample was stored at 4 °C until use. Phages were first enriched in a 96-well microtiter plate and then isolated by the double-layer agar method [22,31]. The individual plaque was picked from the double-layer agar plate, resuspended with 2 mL of TM buffer (20 mM Tris-Cl and 10 mM MgSO_4_), and then used in another round of phage purification using the double-layer agar method. The S-CREM1 isolate was obtained after five rounds of double-layer agar purification.

### 2.2. Host Range Determination

The cross infectivity of S-CREM1 was tested using 11 *Synechococcus* strains: CB0101, A10-1-5-1, CBW1003, CBW1006, CBW1107, CBW1004, PCC 7002, CC9311, WH 8102, WH 7803, and WH 7805. These 11 *Synechococcus* strains were cultured in different mediums with salinities ranging from 15 to 35. Ten microliters of 0.22 µm filtered phage lysates were added to 0.2 mL of exponentially growing *Synechococcus* cultures in a 96-well microtiter plate in triplicate, while control cultures only received SN medium. All plates were incubated in the illumination incubator at 22 °C under 20 µmol photons m^−2^ s^−1^ continuous white light. The infectivity was observed by comparing the cell lysis of the phage-added and the control groups within two weeks.

### 2.3. One-Step Growth Curve

At a multiplicity of infection (MOI) of 0.01, the S-CREM1 were inoculated into 1 mL exponentially growing cultures of *Synechococcus* sp. CB0101 (OD_750_ = 0.5) and incubated for 1 h at 22 °C under 20 µmol photons m^−2^ s^−1^ continuous light for phage adsorption to host cells. The unabsorbed phages were removed by centrifugation at 6000× *g* for 10 min. Precipitated cells were resuspended in 100 mL of fresh SN15 medium in triplicates and incubated under the same conditions described above. Subsamples were taken at 0 h, 4 h, 8 h, 10 h, 12 h, 14 h, 16 h, 20 h, and 24 h to determine the variation in phage concentration. Phage concentration was quantified by quantitative real-time PCR (qPCR) [32], using the portal protein gene (*g20*) as the marker gene. The primers F (5′-TTATGAGTATGCTTGAGGAC-3′) and R (5′-ATGAAGGAACGTTGAGTG-3′) used in the *g20* quantification were designed using the Primer Premier 5 software. The qPCR reactions were performed in a 10 μL qPCR mix, which contained 5 μL of SYBR Premix Ex Taq™ II, 1 μL of each primer, 1 μL of nuclease-free water, and 2 μL of DNA template. Thermal cycling was conducted in a CFX Connect (TM) real-time PCR system (Bio-Rad Laboratories, Hercules, CA, USA) consisting of a 10 min denaturation at 94 °C, and 40 cycles of denaturation at 94 °C for 30 s, annealing at 48 °C for 30 s, and elongation at 72 °C for 30 s.

### 2.4. Phage Amplification and Purification

S-CREM1 phage suspensions were inoculated into 2 L of exponentially growing cultures of *Synechococcus* sp. CB0101 at an MOI of 0.1. After host cell lysis, RNase A and DNase I were added to the lysates both at a final concentration of 2 µg mL^−1^, and they were treated at room temperature for 1 h. Afterward, the NaCl concentration of phage lysates was adjusted to 1 M, and the lysates were ice-bathed for 0.5 h. To remove the remaining cells and debris, the phage lysates were centrifuged at 12,000× *g* at 4 °C for 20 min and further filtered through 0.22 µm filters (Millipore, Bedford, MA, USA). The filtrates were treated with PEG8000 (*w/v* 10%) and kept at 4 °C for 24 h [22,33]. The PEG-treated phage suspensions were centrifuged at 12,000× *g* at 4 °C for 1 h to precipitate phage particles and then resuspended with 6 mL of TM buffer. Concentrated phage particles were then purified by CsCl density gradient ultracentrifugation (gradient density 1.45, 1.5, 1.55, and 1.6 g mL^−1^, 200,000× *g* at 4 °C, 6 h) in a SW 41Ti rotor (Beckman Optima L-100XP, Beckman Coulter, CA, USA) [8,34]. The visible phage band was extracted and then desalted using a 30 kDa centrifugal ultrafiltration unit. The purified high-titer phages were stored at 4 °C.

### 2.5. Transmission Electron Microscopy (TEM) Observation

The carbon-coated copper grids (200-mesh) were subjected to glow discharge for 20 s. Ten microliters of purified S-CREM1 suspensions were adsorbed to carbon-coated copper grids for 5 min and negatively stained twice with 2% (*w/v*) uranyl acetate for 10 s and 30 s, respectively. The stained sample was dried for 10 min and observed using a Tecnai G2 Spirit BioTwin transmission electron microscope (FEI Tecnai G2 F20, Thermo Fisher Scientific, Waltham, MA, USA).

### 2.6. Phage Genome DNA Extraction and Sequencing

Phage DNA was extracted from the purified high-titer phage suspension using the phenol–chloroform method described previously [6,33]. The genomic DNA was sequenced using the Illumina HiSeq 4000 platform by Shanghai Majorbio Bio-pharm Technology Co., Ltd. After quality control and trimming, a total of 2,192,371,253 bp clean reads were obtained. Afterward, the clean reads were assembled using IDBA-UD version 1.1.1 to generate the final complete genome sequence [35].

### 2.7. Genomic and Phylogenetic Analyses

Putative open reading frames (ORFs) of S-CREM1 were predicted by the RAST (http://rast.nmpdr.org/ (accessed on 12 May 2021)), the Gene-MarkS online server (http://exon.gatech.edu/GeneMark/ (accessed on 12 May 2021)), and the Meta Gene Annotator (http://metagene.nig.ac.jp/ (accessed on 12 May 2021)). ORFs of S-CREM1 were annotated using BLASTP search against the NCBI non-redundant (NR) database (e-value < 10^−3^) and conserved domain search against the NCBI Conserved Domain Database (e-value < 10^−3^, bitscore > 40). For S-CREM1 ORFs with no predicted functions based on sequence analyses, distant homolog searches using HHpred (probability > 90%) and Phyre2 (confidence > 80%) were performed to assist the annotation based on predicted structural properties [36,37]. The tRNA genes were predicted by tRNAscan-SE (http://lowelab.ucsc.edu/tRNAscan-SE (accessed on 10 July 2021)) [38]. Other non-coding RNA genes, such as small RNA (sRNA) and *cis*-regulatory RNA genes, were predicted by searching against the Rfam database (https://rfam.xfam.org/family/RF03085 (accessed on 17 September 2022)) [18]. To characterize the genomic similarities of S-CREM1 with other phages, a total of 11,510 viral genomes were downloaded from the NCBI Viral RefSeq database. The similarity score between each pair of viral genomes was calculated by vConTACT 2.0 [39]. The 20 cyanophages that are most closely related to S-CREM1 were selected and further characterized with aspect to the genomic nucleotide similarity using VIRIDIC (http://rhea.icbm.uni-oldenburg.de/VIRIDIC/ (accessed on 14 January 2022)) [40]. Phylogenomic analyses of S-CREM1 and 45 T4-like cyanophages were performed based on amino acid sequences of 30 core genes. The core genes among the 46 phages were identified by OrthoFinder, aligned by MAFFT, and trimmed by TrimAI [41,42,43]. The phylogenomic tree was conducted with RAxML (version 8) employing the maximum likelihood method with the PROTGAMMAJTT model (bootstrap replicates = 100) [44]. Phylogenetic analyses of 2-oxoglutarate (2OG)-Fe(II) oxygenase, MoxR ATPase, and pyrimidine dimer DNA glycosylase genes were performed using the MEGA 7.0 software package [45]. The maximum likelihood method with the Jones–Taylor–Thornton (JTT) model and the neighbor-joining method with the *p*-distance model were used in the phylogenetic tree construction with 1000 bootstrap replicates.

### 2.8. Identification of the S-CREM1 Virion Proteins by Mass Spectrometry

Proteomic analysis of the S-CREM1 virions was performed with CsCl-purified phage suspensions. Fifty microliters of phage suspensions were mixed with the same volume of SDT lysis buffer (4% SDS, 100 mM Tris-HCl, 1 mM dithiothreitol, pH 7.6) and incubated in boiling water for 10 min. Dithiothreitol was added into the suspension at a final concentration of 100 mM and incubated in boiling water for 5 min. Then, 200 µL of UA buffer (8 M urea, 150 mM Tris-HCl, pH 8.0) was added into the suspension, and the detergent was removed through ultrafiltration. A total of 100 µL of iodoacetamide (IAA) buffer (100 mM IAA in UA) was used to modify the UA-buffered sample for 30 min at 25 °C in the dark. The protein suspension was combined with 100 µL of UA buffer and centrifuged at 14,000× *g* for 15 min twice; then, 100 mL of 25 mM NH_4_HCO_3_ was added and the suspension was centrifuged at 14,000× *g* for 15 min twice. Afterward, the protein suspension was digested with 40 µL of trypsin buffer (2 µg of trypsin in 40 µL of 100 mM NH_4_HCO_3_) at 37 °C for 18 h. The tryptic peptides were analyzed using liquid chromatography–electrospray ionization–tandem mass spectrometry (LC-ESI-MS/MS) by Shanghai Applied Protein Technology Co., Ltd. The determination was performed on the Q-Exactive mass spectrometer (Thermo Fisher Scientific, Waltham, MA, USA) that was connected to an Easy nLC (Thermo Fisher Scientific, Waltham, MA, USA). Peptides were fractionated by buffer A (0.1% aqueous formic acid) and buffer B (84% acetonitrile and 0.1% aqueous formic acid) using a C_18_ reversed-phase analytical column (Thermo Fisher scientific EASY column). The custom composite protein database was established based on the S-CREM1 ORF amino acid sequences, and the Mascot 2.4 software (Matrix Science, London, UK) was used to search against the database to analyze the mass spectrometry (MS) data.

### 2.9. Codon Usage (CU) and Relative Synonymous Codon Usage (RSCU) Analyses

To evaluate the potential contribution of tRNA genes to the phage gene translation efficiency, the CUs of S-CREM1 and *Synechococcus* sp. CB0101 were analyzed using Countcodon v4 (http://www.kazusa.or.jp/codon/countcodon.html (accessed on 10 October 2022)). In addition, the RSCU analysis was performed to investigate whether the tRNAs of S-CREM1 and *Synechococcus* sp. CB0101 matched the most used codons in their genomes. The RSCU value is the ratio of the usage frequency of a specific codon to all expected synonymous codons in amino acid synonymous codons [46]. RSCU values >1 or <1 indicate that the CU frequency is higher or lower than expected. The RSCU values of S-CREM1 and *Synechococcus* sp. CB0101 were calculated using the CodonW v1.4.2 software (https://sourceforge.net/projects/codonw/ (accessed on 18 October 2022)).

### 2.10. Motifs Prediction of sRNA and cis-Regulatory RNA Genes

DNA sequence conserved overlapping motifs of sRNA and *cis*-regulatory RNA genes were predicted by MEME Suite 5.5.0 (https://meme-suite.org/meme/doc/meme.html (accessed on 10 October 2022)) [47]. A total of 145 sequences of the *abiF* sRNA identified with solid bit scores (>40) in the Rfam database were selected for the sRNA motif analysis. The sequences of *wcaG*, *manA,* and *glnA cis*-regulatory RNAs identified in cyanophage genomes were selected for conserved motif analyses.

## 3. Results and Discussion

### 3.1. General Features of S-CREM1

**Figure 1:**
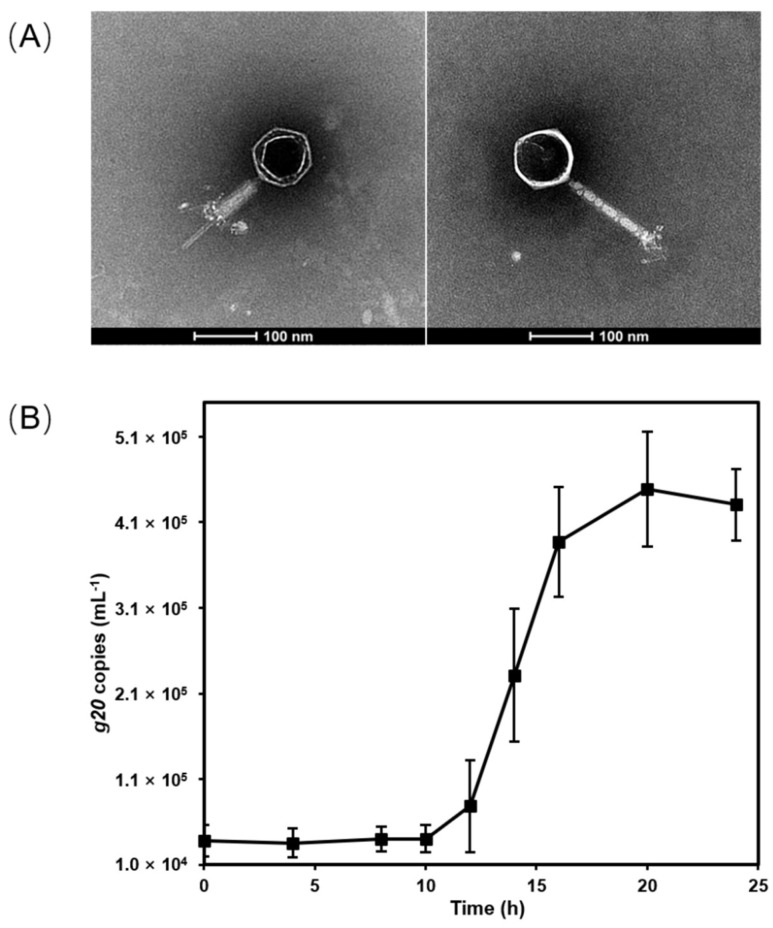
The morphology and growth of S-CREM1. (**A**) Transmission electron microscopy images of S-CREM1 with a contractile sheath (**left**) and a complete tail (**right**). (**B**) One-step growth curve of S-CREM1.

Cyanophage S-CREM1 which infects *Synechococcus* sp. CB0101 was isolated from the surface seawater of the Changjiang River Estuary (30.8°N, 122.6°E) in July 2019 [21]. S-CREM1 is a myovirus with an isometric icosahedral head (approximately 94 nm in diameter) and a contractile tail (approximately 165 nm in length and 13 nm in width) (Figure 1). The one-step growth curve shows that S-CREM1 has a latent period of 10–12 h and a burst size of 11 (Figure 1). Unlike most of the previously identified cyanomyoviruses that usually had a broad host range [4,48], S-CREM1 only infected the original host strain CB0101, while it had no infectivity on other tested *Synechococcus* strains isolated from similar or distinct environments as CB0101 (Table 1). The *Synechococcus* strains used for the host range test include estuarine, coastal and oceanic isolates. The cross-infectivity of S-CREM1 resembles that of another cyanophage S-SZBM1, which also has a narrow host range [49].

### 3.2. Genomic Features of S-CREM1 and Proposal of a New Viral Genus

**Figure 2:**
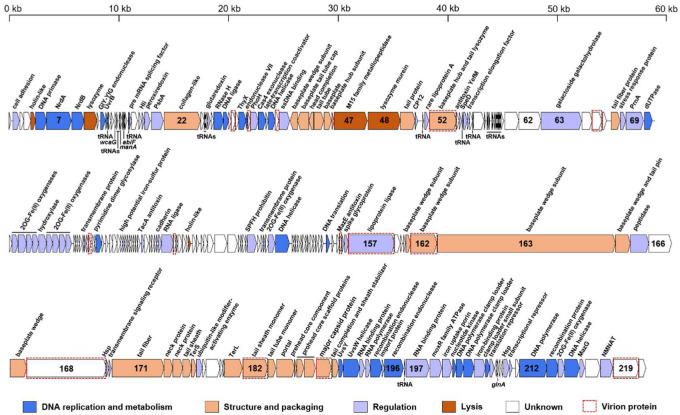
Genome organization of S-CREM1. ORFs with different predicted functional categories are shown in different colors. The direction of ORF transcription is indicated by an arrow. The tRNA, sRNA, and *cis*-regulatory RNA genes are marked underneath the ORF bar. The acronym of GIY-YIG stands for GlyIleTyr–TyrIleGly; ssDNA, single-stranded DNA; 2OG, 2-oxoglutarate; NMNAT, nicotinamide/nicotinate mononucleotide adenylyltransferase. Virion proteins detected in the virion proteome by mass spectrometry analysis are indicated by red dashed frames.

**Figure 3:**
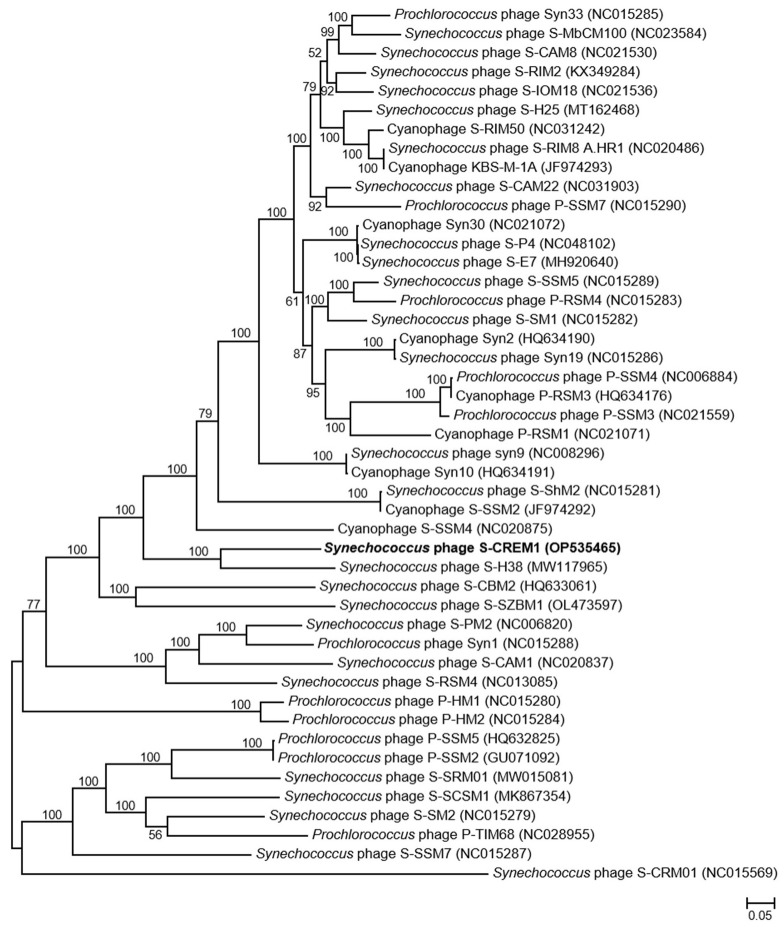
The maximum-likelihood phylogenomic tree based on the 30 core genes among S-CREM1 and 45 T4-like cyanophages. Bootstrap values are calculated based on 100 replicates (only values > 50% are shown).

The genome of S-CREM1 is assembled into a circularly permuted DNA molecule, with a length of 177,957 bp and G + C content of 39.7%. A total of 220 open reading frames (ORFs), 24 tRNA genes, one small RNA (sRNA) gene, and three *cis*-regulatory RNA genes are predicted in the genome of S-CREM1 (Figure 2, Tables S1 and S2). Among the 220 ORFs, 114 ORFs have predictable functions, and 27 ORFs have no homologs in the NR database. The 114 ORFs with predictable functions in S-CREM1 can be divided into four categories, i.e., DNA replication and metabolism (26 ORFs), structure and packaging (29 ORFs), regulation (54 ORFs), and lysis (five ORFs) (Figure 2), accounting for 13%, 29%, 21.5%, and 4.4% of the genome size, respectively. Among the 220 ORFs of S-CREM1, 185 ORFs are homologous to those of T4-like cyanophages, suggesting that S-CREM1 is a member of T4-like cyanophages (Table S1). The phylogenomic analysis among S-CREM1 and 45 T4-like cyanophages based on the 30 core genes showed that S-CREM1 formed a new clade with *Synechococcus* phage S-H38 which was isolated from the Yellow Sea, China (Figure 3). A total of 14 S-CREM1-encoded proteins were detected in the virion proteome by mass spectrometry. Of the 14 phage proteins, four were related to viral structure, including the baseplate, major capsid, and tail proteins (Figure 2). Of the ten remaining proteins, nine have unknown functions and one has no matches in the NR database (Table S1). ORF157 is predicted to be a distant homolog of lipoprotein lipase (Table S2). Lipoprotein lipase is known to play an important role in systemic lipid partitioning and metabolism [54], which may be involved in the conversion of triacylglycerol to diacylglycerol in host glycerolipid metabolism. Since this protein is unlikely to be a structural protein, it could be a highly expressed functional protein carried by the virions. Functional proteins encapsulated by virions have been reported in some cyanophages, which may be important for cyanophage infection [8,22,49].

**Figure 4:**
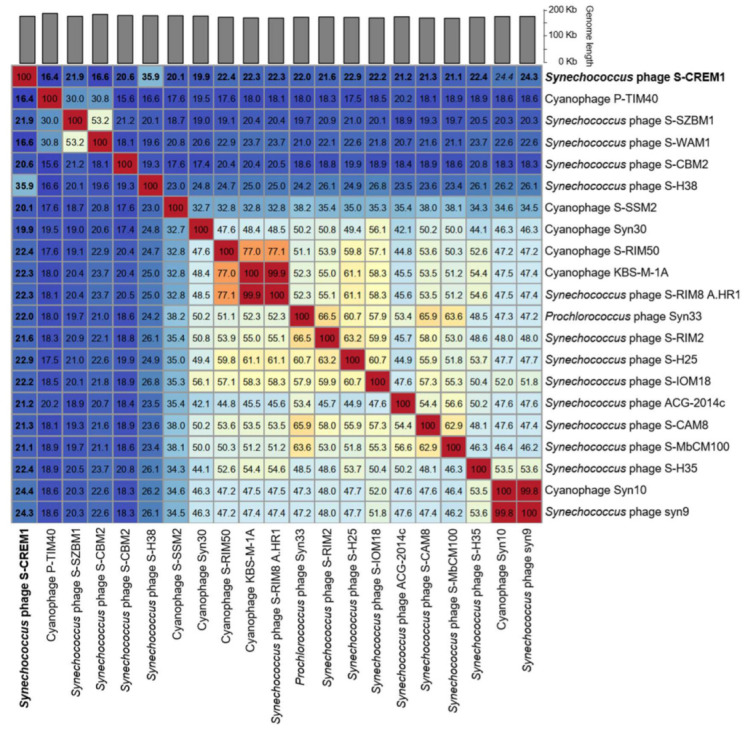
Intergenomic similarities among S-CREM1 and 20 cyanophages calculated using VIRIDIC. Genomic similarities between S-CREM1 and the 20 cyanophages are displayed in bold.

The genomic nucleotide sequence similarities calculated by VIRIDIC between S-CREM1 and the most closely related 20 cyanophages in the NCBI Viral RefSeq database were 19.9–35.9% (Figure 4). According to the recognized virus naming and classification guide, the same genus viruses should share >50% nucleotide sequence similarity [55]. Therefore, we propose that S-CREM1 represents a new cyanophage genus and name it *Symyovirus*. The new genus *Symyovirus* has been submitted to ICTV.

### 3.3. Various and Unique AMGs in the S-CREM1 Genome

#### 3.3.1. Cyanophage-Featured AMGs

The AMGs shared by most cyanophages are present in the S-CREM1 genome, such as photosynthesis-related genes *hli* (ORF19), *psbA* (ORF21), and *speD* (ORF55), carbon metabolism-related gene CP12 (ORF50), and phosphorus-acquisition-related genes *phoH* (ORF35) and *mazG* (ORF216). These phage-encoded photosynthesis-related genes may maintain and enhance the host photosynthetic activity during the viral infection, thus providing a fitness advantage for viral replication and production [9,56]. Protein CP12 is an inhibitor of the Calvin cycle, and the expression of phage CP12 during infection will inhibit the Calvin cycle of the host and direct carbon flux from glucose synthesis to the pentose phosphate pathway, which will retain ATP and NADPH for the viral replication process [10,57]. In addition, S-CREM1 also encodes a tryptophan halogenase (PrnA) that is commonly found in other cyanophage genomes [7]. PrnA (ORF69) catalyzes free tryptophan to chlorotryptophan, which is the first step of antibiotic pyrrolnitrin biosynthesis [58], and it may provide antibiotic protection to the host during S-CREM1 infection. The S-CREM1 genome contains ten ORFs encoding 2OG-Fe(II) oxygenase superfamily proteins (Table S1 and S2), which are predicted to function in DNA repair, protein modification, and lipid metabolism [59]. Moreover, 2OG-Fe(II) oxygenase superfamily proteins are widely distributed in cyanophage genomes, usually ranging from one to five in number [7]. In particular, 24 2OG-Fe(II) oxygenase superfamily protein genes belonging to four subfamilies were predicted in the genome of cyanophage S-SCSM1 [8]. The ten 2OG-Fe(II) oxygenase superfamily protein genes of S-CREM1 can be divided into three subfamilies based on the conserved domains, TIGR02466, pfam13759, and pfam13640, and they show low amino acid sequence identity (0–41.2%) with each other (Table S3, Figure S1). In addition, the ten ORFs clustered into nine clades with cyanophage or heterotrophic bacterial sequences in the phylogenetic analyses (Figure S1), indicating their divergence and potential diverse functions in reprogramming host metabolisms during phage infection.

#### 3.3.2. Three Antitoxin Genes

Notably, the S-CREM1 genome encodes three antitoxin genes, YefM (ORF54), TacA (ORF106), and MazE (ORF155) (Table 2). Toxin–antitoxin (TA) systems are genetic modules consisting of a stable toxin and an unstable antitoxin, which are widespread in many bacteria [60]. Toxin and antitoxin generally exist in pairs, in which toxin may inhibit cell growth or cause cell death, while antitoxin forms stable complexes with a toxin to prevent the toxin from exerting toxicity [61]. TA systems have been proven to play critical roles in protecting bacteria against phage infections [60]. Phage infection will lead to the release of toxins from some TA systems, which can kill the host cell and inhibit phage replication [60]. In addition, the TA system is associated with stress responses in microbes and is a successful survival strategy under various environmental stresses [62]. Many more TA systems are found in freshwater and coastal *Synechococcus* genomes than in open ocean *Synechococcus* strains [63].

The host strain, *Synechococcus* sp. CB0101, was isolated from the Chesapeake Bay [30]. The genome sequencing of CB0101 led to a discovery of wide presence of TA systems in *Synechococcus* [63,64]. The enrichment of TA genes in freshwater and estuarine *Synechococcus* compared to the coastal and ocean counterparts suggests that the inheritance of TA genes helps *Synechococcus* better adapt to changing environments and resist the infection of cyanophages [62,63]. The two Type II TA Pairs, YefM–YoeB and MazE–MazF, are present and expressed in host CB0101 [62], while YefM and MazE in S-CREM1 and host CB0101 have no homology. MazE–MazF is the first TA system described as capable of regulating or causing programmed bacterial death [65]. Phage encoding antitoxin or antitoxin mimics may be the simplest way to overcome TA-mediated defense [60]. Encoding antitoxin genes (YoeB, MazF, and TacA) by phage S-CREM1 may be a mechanism to counteract the TA system of CB0101, which has the potential to reduce the virulent infection to a certain degree so that host cells can survive longer on behalf of phage. It is plausible that antitoxins encoded by S-CREM1 may bind to free toxins in host cells during infection, preventing host cells from being killed to facilitate the replication of S-CREM1. Interestingly, a pair of type II TA genes was found in cyanophage vB_AphaS-CL131, which infects filamentous diazotrophic cyanobacterium and was predicted to play a role in preventing the host from entering dormancy and ensuring the continuous replication of phages [66]. The role of phage-encoded antitoxin genes is interesting and should warrant further investigation.

#### 3.3.3. A MoxR Family ATPase Gene

The S-CREM1 genome carries a gene (ORF198) encoding a homolog of MoxR family ATPase, which is widely found in various prokaryotic species [67,68]. MoxR proteins are important regulators of multiple stress response pathways and are proven to function under acid, oxidative, and heat stresses in different heterotrophic bacteria [69,70]. In addition, the MoxR ATPase has also been found to function as a chaperone and play a role in tail development of *Acidianus* two-tailed virus [71]. The MoxR ATPases are classified into seven major subfamilies: MRP, CGN, APE2220, PA2707, RavA, TM0930, and YehL [68]. The S-CREM1 MoxR ATPase clustered into the CGN subfamily with other cyanophages, bacteriophage, and heterotrophic bacteria, while the cyanobacterial MoxR ATPases fell into the MRP subfamily in the phylogenetic analyses (Figure S2), indicating the different MoxR ATPase gene pool of evolution between cyanophages and their hosts.

#### 3.3.4. Overlooked Pyrimidine Dimer DNA Glycosylase Genes in Cyanophages

S-CREM1 ORF91 is predicted to be a pyrimidine dimer DNA glycosylase by searching the Conserved Domain database. The pyrimidine dimer induced by ultraviolet (UV) is the most common mechanism causing DNA damage in microbes [72]. Pyrimidine dimer DNA glycosylase functions as a base excision repair protein by digesting the pyrimidine dimer of the damaged DNA molecule through hydrolyzing the glycosylic bond of the 5′ pyrimidine and the phosphodiester bond of intra-pyrimidine [73,74,75]. Although pyrimidine dimer DNA glycosylases are frequently found in bacteriophages, only a few pyrimidine dimer DNA glycosylases have been found in isolated cyanophages. There are many homologous sequences of ORF91 in the NCBI NR database (amino acid identity 55.4–64.6%), which were previously predicted as hypothetical proteins (Figure S3). The high similarities of the ORF91 homologs predicted in the genomes of cyanophage isolates with pyrimidine dimer DNA glycosylase in the Conserved Domain database showed that these ORFs may be pyrimidine dimer DNA glycosylases that were previously overlooked. Therefore, we suggest that pyrimidine dimer DNA glycosylase is widely encoded in the cyanophage genomes and plays a role in the repair of damaged DNA during infection. The close phylogenetic relationship of the pyrimidine dimer DNA glycosylase gene among cyanophages and heterotrophic bacteria (Figure S3) indicated that the cyanophage pyrimidine dimer DNA glycosylase genes may exchange with homologs from heterotrophic bacteria rather than their cyanobacterial hosts.

### 3.4. A Large Number of tRNA Genes

Twenty-four bona fide tRNA genes were identified in the S-CREM1 genome, including all but tRNA^Cys^ amino acid specificities (Table S4). Although tRNA genes are frequently found in cyanophage genomes, only S-CREM1 and three cyanophages, S-PM2, S-CBWM1, and S-CRM01, contain more than 20 tRNA genes (Table S5) [22,23,24]. S-CRM01 and S-CBWM1 contain a full set of tRNAs, and S-PM2 only lacks tRNA genes for Cys and Phe [22,23,24]. With the exception of the tRNA gene for the TGA anticodon, S-CREM1 contains the same type of tRNA in the other three cyanophages (Table S6). The presence of a nearly full set of tRNA genes in these four cyanophage genomes could be important to the protein synthesis of both the phage and host (Table S6).

**Figure 5:**
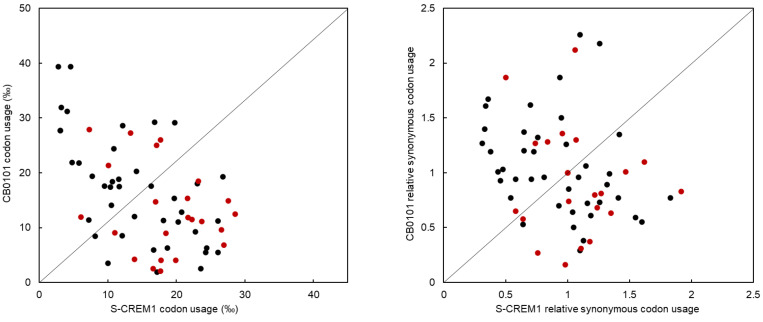
Comparison of CU and RSCU between S-CREM1 and *Synechococcus* sp. CB0101. Each dot represents a codon, and red dots correspond to complementary codons of tRNA genes in S-CREM1.

Phage protein synthesis and reproduction are highly dependent on host transcriptional and translational mechanisms. The reason for phage-carrying tRNAs has always been of interest. A study by Enav et al. revealed that the tRNAs in cyanophages may help to overcome the limitation of cyanophage translation caused by the differences in G + C content among different hosts [15]. In addition, Yang et al. suggest that host tRNA will be degraded upon infection, and then, the tRNA of the phage will supplement the host tRNA function to sustain translation [16]. In addition to the tRNA as anticodon TAT, tRNA genes of the same type as those in S-CREM1 are present in the genome of *Synechococcus* sp. CB0101. If the tRNA genes in S-CREM1 are functional, they may play a more efficient role in supplementing the function of the host tRNAs during phage infection. The comparison of CU and RSCU between S-CREM1 and *Synechococcus* sp. CB0101 showed that S-CREM1 and the host prefer to use different codons (Table 3, Figure 5). Among the 23 codons corresponding to tRNA types shared by S-CREM1 and CB0101, 17 are used more frequently in S-CREM1 than in its *Synechococcus* host, which indicates that S-CREM1 selects and retains tRNAs to compensate for the codon usage difference with its host and facilitate the translation of its own genes [76]. Furthermore, phage tRNAs are found to play roles in regulating translation, packaging, and initiating reverse transcription during infection [77]. It is noteworthy that cyanophages, as well as bacteriophages infecting heterotrophic bacteria with numerous tRNA genes (i.e., >20), are mainly isolated from environments with high nutrient levels (coastal seawater, estuarine water, freshwater, wastewater, and soil) [22]. A large number of tRNA genes in the S-CREM1 genome might be a genomic adaption to the eutrophic environment, enabling S-CREM1 to increase its fitness in the estuary. It would be interesting to know how environmental eutrophication and the growth status of hosts affect the viral possession of tRNA genes. The metabolic activity of prokaryotes is generally higher in the eutrophic environment than that in the oligotrophic habitat, and the expression of phage tRNA genes likely increases the translation efficiency during infection with sufficient nutrients available [22,78].

### 3.5. One Small RNA (sRNA) and Three cis-Regulatory RNA Genes

**Figure 6:**
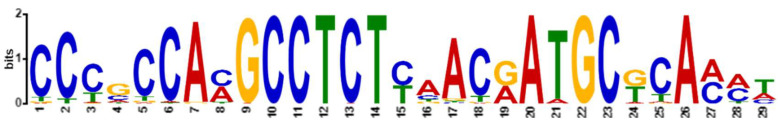
The DNA sequence conserved motif in the *abiF* RNA genes among S-CREM1 and 139 sequences identified from 136 bacterial and three bacteriophage genomes in the Rfam database.

One small RNA (sRNA) and three *cis*-regulatory RNA genes were identified in the S-CREM1 genome by searching against the Rfam database (Table 4). As major regulatory molecules in bacteria, sRNAs and *cis*-regulatory RNAs play important roles in nutrient uptake and metabolism [79], iron regulation [80], protein synthesis, RNA processing [17], biofilm matrix formation [81], and quorum sensing [82]. The *abiF* sRNA predicted in the S-CREM1 genome was not previously identified in cyanophage genomes in the Rfam database. The S-CREM1 *abiF* gene shares a conserved motif with 139 *abiF* genes that are identified from a variety of bacteria and three bacteriophage genomes in the Rfam database (Figure 6), indicating the high conservation of the *abiF* sRNA gene among microbial organisms and the high possibility that the S-CREM1 *abiF* sRNA is functional during phage infection. The three *cis*-regulatory elements predicted in the S-CREM1 genome are *wcaG*, *manA,* and *glnA*, which were also identified in other cyanophage genomes (Table S7). The *wcaG*, *manA* and *glnA* genes between S-CREM1 and other cyanophages share similar patterns of conserved motifs (Figure S4). *cis*-regulatory elements function as environmental change detectors, such as light or temperature variations, and regulate the message stability or translational efficiency of specific genes. The *wcaG* RNA may regulate the expression of genes related to the production of exopolysaccharides [17]. The *manA* RNA domains are usually located in the potential 5′ untranslated regions of the genes related to nucleotide synthesis, mannose or fructose metabolism, and photosynthesis. Whether the *manA* RNA plays a regulatory role in the expression of these genes remains to be further verified [17]. The *glnA* RNA can regulate the expression of genes related to nitrogen metabolism, such as genes encoding nitrogen regulatory protein PII, glutamine synthetase, glutamate synthase, and ammonium transporters [17,83].

sRNA and *cis*-regulatory RNA have been frequently studied in bacteria and archaea. However, little is known about the types and functions of those regulatory elements in phages, especially in cyanophages. The phage–host system of S-CREM1-*Synechococcus* sp. CB0101 provides a good model for future research on the regulatory functions of phage-encoded sRNAs and *cis*-regulatory RNAs during viral infection.

## 4. Conclusions

Based on the phylogenomic analysis and comparative genomics, we proposed a new genus, *Symyovirus*, for cyanophage S-CREM1 which infects an estuarine *Synechococcus*. S-CREM1 exhibits several interesting genetic features including the possession of three antitoxin genes, the MoxR family ATPase and the pyrimidine dimer DNA glycosylase genes. We reported the presence of three antitoxin genes in the S-CREM1 genome and proposed a potential beneficial role of having antitoxin genes in cyanophages. The presence of a large number of tRNA genes suggests that S-CREM1 may have the capability to thrive in a nutrient-rich estuarine environment. The sRNA gene and three *cis*-regulatory RNA genes suggest that S-CREM1 has other functions in regulating host metabolism during infection. The isolation of cyanophage S-CREM1 and its genomic characterization provide new insights into phage taxonomy, evolution, and phage–host interactions.

## Figures and Tables

**Table 1:** Host range of cyanophage S-CREM1.

Tested Strain	Phylogenetic Clade	Isolation Source	Medium Salinity	Reference	Result ^a^
Estuarine strains					
*Synechococcus* sp. CB0101	CB4, subcluster 5.2	Chesapeake Bay	15	[21]	+
*Synechococcus* sp. A10-1-5-1	CB5, subcluster 5.2	Changjiang River Estuary	25	Xu et al. unpublished	−
*Synechococcus* sp. CBW1003	Bornholm Sea	Chesapeake Bay	15	[50]	−
*Synechococcus* sp. CBW1006	Bornholm Sea	Chesapeake Bay	15	[50]	−
*Synechococcus* sp. CBW1107	Subalpine C II	Chesapeake Bay	15	[50]	−
*Synechococcus* sp. CBW1004	Unclassified	Chesapeake Bay	15	[50]	−
*Synechococcus* sp. PCC 7002	Unclassified	Magueyes Island	22	[51]	−
Marine strains					
*Synechococcus* sp. CC9311	I, subcluster 5.1	California Current	35	[52]	−
*Synechococcus* sp. WH 8102	III, subcluster 5.1	Tropical Atlantic	30	[53]	−
*Synechococcus* sp. WH 7803	V, subcluster 5.1	Sargasso Sea	15	[29]	−
*Synechococcus* sp. WH 7805	VI, subcluster 5.1	Sargasso Sea	35	[29]	−

^a^ +, infected; −, uninfected.

**Table 2:** Three antitoxin genes predicted in the S-CREM1 genome.

ORF No.	Antitoxin	TA System	TA System in the Host Genome
54	YefM	YefM–YoeB	*+*
106	TacA	TacA–TacT	*−*
155	MazE	MazE–MazF	*+*

**Table 3:** CU and RSCU in S-CREM1 and *Synechococcus* sp. CB0101.

Codon ^a^	Attribute	Usage (‰)		RSCU		Codon ^a^	Attribute	Usage (‰)		RSCU		Codon ^a^	Attribute	Usage (‰)		RSCU		Codon ^a^	Attribute	Usage (‰)		RSCU	
		Phage	Host	Phage	Host			Phage	Host	Phage	Host			Phage	Host	Phage	Host			Phage	Host	Phage	Host
TTT	Phe	18.7	6.3	0.9	0.7	TCT	Ser	22.8	9.2	1.6	0.6	TAT	Tyr	23.6	2.5	1.3	0.7	TGT	Cys	12.1	8.5	1.1	0.5
**TTC**	Phe	21.7	11.8	1.1	1.3	TCC	Ser	10.5	14.1	0.7	0.9	**TAC**	Tyr	13.9	4.2	0.7	1.3	TGC	Cys	10.9	24.4	1	1.5
**TTA**	Leu	17.7	2.1	1	0.2	**TCA**	Ser	21.6	15.3	1.5	1	TAA	Stop	17.2	1.9	1.1	0.3	TGA	Stop	19.7	15.3	1.3	2.2
TTG	Leu	20.8	12.8	1.2	1.1	TCG	Ser	9.5	17.6	0.7	1.2	TAG	Stop	10	3.5	0.6	0.5	**TGG**	Trp	17.1	25	1	1
CTT	Leu	18.1	11.3	1	0.9	CCT	Pro	11.6	18.8	1.4	0.8	CAT	His	13.9	12	1.2	0.7	**CGT**	Arg	6.1	11.9	0.6	0.6
CTC	Leu	11.7	17.5	0.7	1.4	CCC	Pro	4.8	21.9	0.6	0.9	**CAC**	His	10.1	21.3	0.8	1.3	CGC	Arg	3.2	31.9	0.3	1.6
**CTA**	Leu	19.9	4	1.1	0.3	**CCA**	Pro	13.3	27.3	1.6	1.1	**CAA**	Gln	27.6	14.9	1.2	0.7	CGA	Arg	7.7	19.4	0.8	1
CTG	Leu	19.8	29.1	1.1	2.3	CCG	Pro	3.1	27.7	0.4	1.2	CAG	Gln	16.8	29.2	0.8	1.3	CGG	Arg	3.1	27.7	0.3	1.4
ATT	Ile	26.1	5.5	1.2	0.6	ACT	Thr	24.4	6.3	1.6	0.6	AAT	Asn	24.3	5.5	1	0.6	AGT	Ser	16.7	5.9	1.1	0.4
**ATC**	Ile	23.2	18.5	1.1	2.1	ACC	Thr	10.7	18.4	0.7	1.6	**AAC**	Asn	22.3	11.5	1	1.4	**AGC**	Ser	7.3	27.9	0.5	1.9
ATA	Ile	16.6	2.5	0.8	0.3	**ACA**	Thr	18.5	9	1.2	0.8	AAA	Lys	26.9	6.8	1	0.7	**AGA**	Arg	26.6	9.6	2.8	0.5
**ATG**	Met	23.7	11.1	1	1	ACG	Thr	7.2	11.4	0.5	1	AAG	Lys	26.1	11.2	1	1.3	AGG	Arg	10.4	17.4	1.1	1
GTT	Val	20.3	11	1.3	1	GCT	Ala	12.2	28.6	1.3	0.9	GAT	Asp	26.8	19.3	1.4	1.4	GGT	Gly	23.1	18	1.8	0.8
GTC	Val	8.2	8.4	0.5	0.8	GCC	Ala	2.8	39.4	0.3	1.3	**GAC**	Asp	11	9.1	0.6	0.7	GGC	Gly	4.6	39.4	0.4	1.7
**GTA**	Val	17.8	4	1.2	0.4	**GCA**	Ala	17.7	26	1.9	0.8	**GAA**	Glu	28.6	12.5	1.3	0.8	**GGA**	Gly	17	14.7	1.4	0.6
GTG	Val	14.2	20.3	0.9	1.9	GCG	Ala	4.1	31.2	0.4	1	GAG	Glu	16.3	17.6	0.7	1.2	GGG	Gly	5.8	21.8	0.5	0.9

^a^ tRNA genes in S-CREM1 are denoted by their complementary codons in red font, and tRNA genes in *Synechococcus* sp. CB0101 are denoted by their complementary codons in black bold font.

**Table 4:** One sRNA and three *cis*-regulatory RNA genes in the S-CREM1 genome.

Feature	Type	Strand	Start	End	Rfam Accession No.	Score	E-Value
*abiF*	sRNA	+	10531	10567	RF03085	51.6	3.2E-06
*wcaG*	*cis*-regulatory	+	9745	9842	RF01761	91.4	2.3E-15
*manA*	*cis*-regulatory	+	10215	10429	RF01745	100.6	4.6E-23
*glnA*	*cis*-regulatory	−	164330	164232	RF01739	44.0	5.2E-06

## Data Availability

The complete genome sequence of *Synechococcus* phage S-CREM1 was submitted to the GenBank database under accession number OP535465.

## Supplementary Materials

The following supporting information can be downloaded at: https://www.mdpi.com/article/10.3390/v15020380/s1, Figure S1: Unrooted maximum likelihood phylogenetic tree of the S-CREM1 predicted 2OG-Fe(II) oxygenase superfamily proteins. ORFs in brown font were identified based on predicted structural properties using HHpred and Phyre analyses. Bootstrap values (maximum-likelihood/neighbor-joining) are based on 1000 replicates, and only values > 50% are shown; Figure S2: Unrooted maximum likelihood phylogenetic tree of the MoxR ATPases. The number of bootstrap replicates = 1000. The bootstrap values (maximum-likelihood/neighbor-joining) are shown near each node, and only values >50% are shown; Figure S3: Unrooted maximum-likelihood phylogenetic tree of the S-CREM1 pyrimidine dimer DNA glycosylase. The bootstrap values (maximum-likelihood/neighbor-joining) of >50% are shown near each node. The number of bootstrap replicates = 1000; Figure S4: DNA sequence conserved motifs in *wcaG* (A), *manA* (B), and *glnA* (C) *cis*-regulatory RNA genes among S-CREM1 and other cyanophages in the Rfam database; Table S1: Predicted ORFs in the S-CREM1 genome with homologs in the NCBI non-redundant (NR) database; Table S2: Predicted ORFs in the S-CREM1 genome with distant homologs detected by using HHpred and Phyre2 search; Table S3: Amino acid sequence identities among the S-CREM1 2OG-Fe(II) oxygenase family proteins; Table S4: tRNA genes in the S-CREM1 genome; Table S5: Information of cyanophages with tRNA genes more than twenty; Table S6: Comparison of tRNA complementary codons among cyanophages with tRNA genes more than twenty; Table S7: *wcaG*, *manA*, and *glnA cis*-regulatory RNA genes found in cyanophages.
