# Supplementary material for: An Estuarine Cyanophage S-CREM1 Encodes Three Distinct Antitoxin Genes and a Large Number of Non-Coding RNA Genes
Source: Viruses. 2023 Jan 28;15(2):380. doi: 10.3390/v15020380 (PMC9964418; doi:10.3390/v15020380)
Supplement: Supplementary file 1 [file viruses-15-00380-s001.zip › viruses-2128678-supplementary.pdf]

Table S1. Predicted ORFs in the S-CREM1 genome with homologs in the NCBI non-redundant (NR) database.

ORF	Strand	Left	Right	aa length	Significant hit in NR database[a]	Putative function[b]	E-value	aa identity (%)	Conserved domain (E-value)[c]
1	+	1	174	57	hypothetical protein [*Synechococcus* phage S-H38]		2.3E-14	50.9	
2	+	390	998	202	virion structural protein [uncultured *Mediterranean* phage uvMED]		2.2E-83	69.4	
3	+	1012	1374	120	hypothetical protein [*Synechococcus* phage S-H38]		1.5E-52	65	
4	+	1376	2008	214	hypothetical protein [*Synechococcus* phage S-H38]		2.8E-45	47.5	
5	+	2022	2456	144	DNA primase subunit [*Synechococcus* phage ACG-2014d]		1.5E-16	36.5	
6	+	2453	3451	332	DNA primase subunit [*Synechococcus* phage S-H38]	DNA primase subunit	3.8E-155	63.9	PHA02540 (1.9E-140)
7	+	3448	5760	770	ribonucleotide reductase subunit A [*Synechococcus* phage S-H38]	ribonucleotide reductase subunit A (NrdA)	0	81.8	PHA02572 (0)
8	+	5771	6913	380	ribonucleotide reductase subunit B [*Synechococcus* phage S-H38]	ribonucleotide reductase subunit B (NrdB)	0	85.3	PRK09101 (0)
9	+	6910	8091	393	hypothetical protein [*Synechococcus* phage S-PM2]	lysozyme	1.9E-119	73.1	cd00737 (3.8E-14)
11	+	8416	8847	143	NAD synthetase [*Synechococcus* phage ACG-2014d]	GIY-YIG endonuclease	9.3E-74	74.8	cd10444 (1.7E-14)
12	+	8898	9050	50	hypothetical protein [*Synechococcus* phage S-H38]		1.7E-08	66.7	
13	+	9149	9397	82	hypothetical protein [*Synechococcus*		2.5E-41	77.5	

					phage S-SKS1]				
14	+	9390	9638	82	hypothetical protein [*Synechococcus* phage S-H38]		3.1E-14	63.5	
15	+	10664	10819	51	hypothetical protein [*Synechococcus* phage S-SRM01]		6.9E-07	52.9	
16	+	11050	11250	66	hypothetical protein [*Synechococcus* phage S-RIM2]		1.7E-19	68.9	
17	+	11247	11825	192	hypothetical protein [*Synechococcus* phage S-SRM01]		4.9E-99	73.9	
19	+	12232	12450	72	high light inducible protein [*Synechococcus* phage S-CBP4]	high light inducible protein (Hli)	3.7E-22	60.9	PHA02337 (1.3E-13)
20	+	12454	12963	169	peroxiredoxin [*Synechococcus* phage S-SRM01]	peroxiredoxin	6.4E-69	61.3	COG0678 (3.9E-72)
21	+	13104	14183	359	photosystem II q(b) protein [*Synechococcus* phage S-PM2]	photosystem II q(b) protein (PsbA)	0	95.5	TIGR01151 (0)
22	+	14262	17600	1112	hypothetical protein [*Synechococcus* phage S-H38]		1.5E-73	39.7	
23	+	17614	17922	102	hypothetical protein [*Synechococcus* phage ACG-2014d]		6.2E-07	40	
25	+	18277	18513	78	glutaredoxin [*Synechococcus* phage S-CAM9]	glutaredoxin	1.9E-27	53.9	cd03418 (3.8E-12)
26	+	18585	18827	80	hypothetical protein [*Synechococcus* phage S-H38]		9.7E-23	49.4	
27	+	18833	19708	291	RNase H [*Synechococcus* phage S-H38]	RNase H	1.8E-163	75.3	PHA0256 (2.8E-111)
28	+	19710	20162	150	hypothetical protein [*Synechococcus* phage S-H38]		1.1E-88	82.7	
30	+	20413	20832	139	hypothetical protein [*Synechococcus*		8.8E-41	58.3	

					phage S-SRM01]				
31	+	20804	21091	95	hypothetical protein [Cyanophage S-RIM14]		5.4E-10	37.9	
32	+	21084	21773	229	thymidylate synthase [*Synechococcus* phage S-H25]	thymidylate synthase (ThyX)	3.9E-111	66.1	PRK00847 (1.1E-77)
33	+	21773	21919	48	hypothetical protein [*Synechococcus* phage S-H38]		3.6E-17	72.9	
34	+	21932	22162	76	hypothetical protein [Cyanophage S-RSM1]		1.9E-39	81.6	
35	+	22164	22919	251	phosphate starvation-inducible protein PhoH and related proteins [*Synechococcus* phage S-H38]	phosphate starvation-inducible protein (PhoH)	3.9E-161	83.3	pfam02562 (1.2E-37)
36	+	22919	23593	224	exonuclease [*Synechococcus* phage S-H38]	CRISPR/Cas system-associated protein Cas4	3.7E-115	72.6	cd09637 (4.1E-05)
37	+	23583	23837	84	gp33 late promoter transcription accessory protein [*Synechococcus* phage metaG-MbCM1]	late-transcription coactivator	7.5E-37	75.3	pfam16805 (2.8E-22)
38	+	23834	24445	203	loader of DNA helicase [*Synechococcus* phage S-H38]	DNA helicase	3.3E-91	61.9	PHA02559 (7.3E-60)
39	+	24442	24774	110	hypothetical protein [*Synechococcus* phage S-H38]		7.9E-46	69.2	
40	+	24808	25794	328	single-stranded DNA binding protein [*Synechococcus* phage S-H38]	single-stranded DNA binding protein	5.4E-175	83.2	PHA02550 (2.1E-139)
41	-	26492	25827	221	baseplate wedge subunit [*Synechococcus* phage S-H38]	baseplate wedge subunit	1.6E-47	40.6	PHA02578 (4.1E-10)
42	-	27541	26495	348	baseplate tail tube cap [*Synechococcus* phage S-H38]		1.5E-77	42.5	
43	-	27996	27547	149	head completion protein [*Synechococcus*	head completion protein	9.3E-78	77.1	PHA02552 (6.7E-70)

					phage S-H38]				
44	+	28036	28881	281	structural protein [*Synechococcus* phage S-P4]		4.5E-31	36.9	
45	+	28926	29639	237	baseplate hub subunit [*Synechococcus* phage S-H38]	base plate protein	9.9E-127	70.9	pfam12322 (1.4E-15)
46	+	29669	29842	50	baseplate hub assembly catalyst [*Synechococcus* phage S-RIM8 A.HR1]		1.2E-07	50	
47	+	29842	32892	1016	hypothetical protein [*Synechococcus* phage S-H9-2]	M15 family metallopeptidase	1.4E-51	68.9	cd14814 (1.9E-18)
					M15 family metallopeptidase [*Brevibacterium permense*]		2.2E-26	54.5	
48	+	32965	35940	991	lysozyme murein [*Synechococcus* phage S-CAM22]		4.8E-17	61.6	
49	+	35947	37284	445	structural protein [Cyaphage S-RIM50]		2.4E-62	33.9	
50	+	37312	37524	70	CP12 [uncultured *Mediterranean* phage uvMED]	CP12	7.4E-20	55.4	smart01093 (1.1E-15)
51	+	38173	38535	120	rare lipoprotein A [*Synechococcus* phage S-CBS4]	rare lipoprotein A	8.3E-38	61.5	cd22268 (1.3E-42)
52	+	38604	41090	828	baseplate hub + tail lysozyme [*Synechococcus* phage S-H38]	baseplate hub subunit and tail lysozyme	0	49.2	COG5412 (5.4E-11)
53	+	41112	41339	75	hypothetical protein [*Synechococcus* phage S-H38]		1.3E-14	45.8	
54	+	41336	41587	83	hypothetical protein [*Synechococcus* phage S-H38]		2.7E-17	44.6	
55	+	41746	42096	116	S-adesylmethionine decarboxylase [*Synechococcus* phage S-H38]	adesylmethionine decarboxylase (SpeD)	4.1E-50	66.4	PRK02770 (4E-52)
56	+	42086	42304	72	hypothetical protein [*Synechococcus*		4.4E-06	38.1	

					phage ACG-2014c]				
57	+	42310	42579	89	hypothetical protein [*Synechococcus* phage S-SRM01]		2.7E-39	71.3	
58	+	42670	43620	316	hypothetical protein [Lachnospiraceae bacterium]		1.3E-11	29	
60	+	45198	45419	73	hypothetical protein [Cytophagia bacterium]		1.1E-10	44.3	
61	+	45527	46831	434	YadA domain protein [*Synechococcus* phage ACG-2014c]		2.1E-122	47.1	
62	+	46839	48854	671	structural protein [*Synechococcus* phage S-WAM1]		2.7E-68	32.7	
63	+	48880	52569	1229	tail fiber protein [*Synechococcus* phage S-H38]		0	80.4	
64	+	52601	53536	311	structural protein [*Synechococcus* phage S-E7]		2.3E-30	46.5	
65	+	53542	54438	298	keratin, type I cytoskeletal 9 [*Lingula anatina*]		1.2E-20	47	
66		54494	54862	122	tail fiber protein [*Synechococcus* phage S-WAM1]		5E-13	0.5	
67	+	55301	56104	267	tail fiber protein [*Synechococcus* phage S-WAM1]		6.6E-52	49.1	
68	+	56135	56623	162	gp40 [*Synechococcus* phage syn9]		6.2E-05	56.4	
69	+	56663	58267	534	putative tryptophan halogenase [*Synechococcus* phage syn9]	tryptophan halogenase (PrnA)	0	60.3	pfam04820 (4.8E-105)
70	+	58331	59068	245	putative Deoxyuridine 5'-triphosphate nucleotidohydrolase [uncultured *Mediterranean* phage uvMED]		1E-11	28.8	
71	+	59065	59286	73	gp44 clamp loader subunit [*Synechococcus*		2.7E-29	73.9	

					phage syn9]				
72	+	59290	59889	199	2OG-Fe(II) oxygenase family protein [*Caulobacter* sp. S45]	2OG-Fe(II) oxygenase family protein	1.7E-09	35.9	pfam13759 (6.4E-14)
73	+	59891	60673	260	gp46 [*Synechococcus* phage syn9]		5.1E-89	49.2	
74	+	60670	61209	179	gp47 [*Synechococcus* phage syn9]	2OG-Fe(II) oxygenase family protein	4.9E-75	60.4	pfam13759 (8.3E-05)
					2OG-Fe(II) oxygenase family protein [*Stappia stellulata*]		1.5E-05	27	
75	+	61206	61790	194	2OG-Fe(II) oxygenase superfamily domain containing protein [*Synechococcus* phage S-WAM1]	2OG-Fe(II) oxygenase family protein	5.9E-32	38.1	TIGR02466 (2.5E-31)
76	+	61783	62493	236	JmjC domain containing protein [uncultured Caudovirales phage]	ribosomal protein hydroxylase	2.3E-106	61.2	COG2850 (2.7E-04)
77	+	62486	63052	188	Prolyl 4-hydroxylase alpha subunit homologue [uncultured *Mediterranean* phage uvMED]		4.3E-23	31.4	
79	+	63663	64256	197	gp53 [*Synechococcus* phage syn9]		2.1E-78	59.6	
					2OG-Fe(II) oxygenase [*Betaproteobacteria* bacterium]		1.9E-21	31	
80	+	64253	64780	175	gp54 [*Synechococcus* phage syn9]		5.6E-73	60.8	
					2OG-Fe(II) oxygenase [*Betaproteobacteria* bacterium]		6.7E-27	36.4	
82	+	65149	65313	54	hypothetical protein [*Synechococcus* phage S-H38]		3.3E-09	52.4	
84	+	65488	65739	83	hypothetical protein [*Synechococcus* phage S-H38]		3.3E-12	48.5	

86	+	65930	66115	61	hypothetical protein [Bacterium]		1E-11	54.1	
87	+	66130	66348	72	putative membrane protein [*Synechococcus* sp. ROS8604]		9.6E-06	39.4	
88	+	66351	66557	68	putative 2OG-Fe(II) oxygenase [*Synechococcus* phage S-SCSM1]		3.1E-11	47.6	
89	+	66719	66898	59	hypothetical protein [Bacterium]		6.6E-15	62.8	
91	+	67025	67549	174	hypothetical protein [*Synechococcus* phage ACG-2014f]	pyrimidine dimer DNA glycosylase	1E-78	64	pfam03013 (8.4E-11)
					pyrimidine dimer DNA glycosylase/endonuclease V [*Nonlabens xiamenensis*]		1.2E-66	58.1	
92	+	67546	67737		hypothetical protein [*Synechococcus* phage SynMITS9220M01]		1.2E-06	48.8	
93	+	67881	68111		hypothetical protein [*Synechococcus* phage S-PM2]		1.6E-17	55.6	
94	+	68262	68561		hypothetical protein [*Synechococcus* phage S-SSM7]		4.8E-19	52.7	
95	+	68539	68958		hypothetical protein [*Synechococcus* phage S-CBWM1]		2.1E-08	33.7	
97	+	69257	69451		hypothetical protein [*Synechococcus* phage S-PM2]		1.9E-07	53.1	
98	+	69444	69671		hypothetical protein [*Synechococcus* phage S-SRM01]		2.4E-07	42.4	
100	+	69856	70032	58	hypothetical protein [Bacterium]		1E-03	45.3	
101	+	70055	70240	61	hypothetical protein [*Synechococcus* phage S-SRM01]		4.5E-15	56.9	

103	+	70439	70633	64	hypothetical protein [*Synechococcus* phage B3]		6.3E-09	40.3	
104	+	70630	70836	68	hypothetical protein [*Synechococcus* phage S-SRM01]		1.1E-11	52.3	
105	+	70833	71078	81	hypothetical protein [*Synechococcus* phage S-SRM01]		1.6E-06	34.5	
106	+	71163	71441	103	hypothetical protein [*Synechococcus* phage S-SRM01]		1.3E-18	52	
108	+	71896	72117	73	hypothetical protein [*Synechococcus* phage S-PM2]		4.8E-14	75	
					transmembrane protein [*Siphoviridae* sp.]		1.4E-17	66.1	
109	+	72114	72353	79	hypothetical protein [*Synechococcus* phage S-SRM01]		8.7E-20	60.3	
110	+	72350	72541	63	hypothetical protein [Cyanophage S-RIM50]		8E-13	51.6	
111	+	72538	72717	59	hypothetical protein [*Synechococcus* phage S-SRM01]		7.6E-23	92	
112	+	72714	73007	97	hypothetical protein [*Synechococcus* phage S-SRM01]		4.4E-09	42.4	
113	+	73004	74086	360	RNA ligase [uncultured Caudovirales phage]	RNA ligase	5.7E-120	55.3	PHA02142 (6.2E-78)
114	+	74098	74334	78	hypothetical protein [*Synechococcus* phage S-SRM01]		1.6E-18	59.2	
116	+	74501	74896	131	hypothetical protein [*Synechococcus* phage S-PM2]		1.3E-71	78.3	
117	+	74896	75213	105	hypothetical protein [*Synechococcus* phage ACG-2014f]		4.8E-08	51.2	
118	+	75269	75454	61	hypothetical protein [*Synechococcus*		1.1E-10	63.4	

					phage S-H25]				
121	+	76180	76413	77	hypothetical protein [*Synechococcus* phage S-PM2]		4.2E-24	65.7	
123	+	76849	77211	120	hypothetical protein [*Synechococcus* phage S-H38]		1.5E-17	42.5	
124	+	77211	77753	180	hypothetical protein [*Synechococcus* phage S-CAM9]		2.8E-59	55.8	
125	+	77873	78976	367	hypothetical protein [Cyanophage S-RIM44]		3.6E-130	50.3	
126	+	79042	79953	303	hypothetical protein [*Synechococcus* phage S-H38]		6.1E-112	53.4	
					site-specific DNA-methyltransferase [*Caulobacteraceae* bacterium]		1E-57	41.4	
128	+	80276	80404	42	hypothetical protein [*Synechococcus* phage S-SRM01]		7.8E-12	66.7	
129	+	80413	80685	90	hypothetical protein [*Synechococcus* phage B3]		8.5E-29	59.3	
131	+	80872	81690	272	hypothetical protein [*Synechococcus* phage B3]	SPFH prohibitin	3.4E-116	62.7	pfam01145 (4.7E-16)
					SPFH domain-containing protein [*Nitrospinae* bacterium]		4.4E-25	32.1	
132	+	81780	82016	78	hypothetical protein [*Synechococcus* phage S-CBM2]		1.8E-17	52.6	
133	+	82099	82293	64	hypothetical protein [*Synechococcus* phage S-CBM2]		1.4E-15	50	
134	+	82293	82499	68	putative membrane protein [*Synechococcus* sp. A15-127]		3.5E-07	33.3	

135	+	82496	82771	91	hypothetical protein [*Synechococcus* phage S-SRM01]		6.8E-28	60.5	
136	+	82768	83388	206	hypothetical protein [*Synechococcus* phage S-WAM1]	2OG-Fe(II) oxygenase	1.3E-14	30.5	pfam13759 (1.3E-12)
					2OG-Fe(II) oxygenase family protein [*Stemitos rutilans* HA7619-LM2]		1.7E-09	33.3	
137	+	83426	84685	419	DNA helicase [*Synechococcus* phage S-H38]	DNA helicase	0	78.5	COG1061 (2.1E-22)
138	+	84941	85102	53	hypothetical protein [Cyanophage P-RSM1]		6.1E-08	51	
139	+	85106	85267	53	hypothetical protein [*Synechococcus* phage S-RIM8 A.HR1]		1.5E-26	88.7	
140	+	85271	85468	65	hypothetical protein [Cyanophage S-RIM50]		1.7E-31	86.4	
141	+	85465	85647	60	hypothetical protein [*Synechococcus* phage S-CBM2]		8.8E-11	45.5	
143	+	85896	86174	92	hypothetical protein [Bacterium]		2E-12	37.6	
145	+	86335	86466	43	hypothetical protein [*Synechococcus* phage S-SRM01]		1.5E-04	47.6	
146	+	86596	86850	84	hypothetical protein [*Synechococcus* phage ACG-2014d]		1.1E-27	61.4	
147	+	86879	87094	71	hypothetical protein [*Synechococcus* phage S-CBM2]		7.1E-28	71.8	
149	+	87348	87602	84	hypothetical protein [*Synechococcus* phage Bellamy]		4.9E-08	42.7	
151	+	88076	88393	105	hypothetical protein [*Synechococcus* phage S-CBM2]		7.3E-46	68.8	
155	+	89516	89680	54	AbrB/MazE/SpoVT family DNA-binding domain-containing protein [*Cyabacteria*		4.3E-06	39.6	

					bacterium REEB444]				
156	+	89733	90080	115	hypothetical protein [*Synechococcus* phage S-H38]		1.2E-27	50.9	
157	+	90084	94295	1403	hypothetical protein [*Synechococcus* phage S-H38]		8.1E-89	33.6	
158	+	94295	94816	173	structural protein [*Synechococcus* phage S-P4]		1.1E-09	32.4	
159	+	94863	95003	46	hypothetical protein [*Synechococcus* phage S-H25]		1E-13	73.9	
160	+	95130	95309	59	hypothetical protein [*Synechococcus* phage S-H35]		5.2E-11	56.3	
161	+	95356	95775	139	baseplate wedge subunit [*Synechococcus* phage S-H38]	baseplate wedge subunit	4.3E-38	49.6	PHA00415 (3.4E-37)
162	+	95775	98180	801	baseplate wedge subunit [*Synechococcus* phage S-H38]	baseplate wedge subunit	0	55.8	PHA02553 (9.1E-148)
163	+	98218	114486	5422	baseplate wedge initiator [*Synechococcus* phage S-H38]	baseplate wedge	0	52.9	pfam14240 (2.3E-28)
164	+	114518	115843	441	baseplate wedge subunit and tail pin [*Synechococcus* phage S-H38]	baseplate wedge subunit and tail pin	6.6E-50	39.7	PHA02582 (2.1E-08)
165	+	115845	117524	559	subtilisin-like serine protease [*Synechococcus* phage S-H38]	peptidase S8 family domain	0	58	cd04077 (4.4E-34)
166	+	117529	119637	702	baseplate wedge initiator [*Synechococcus* phage S-H38]		0	60.7	
167	+	119683	121215	510	baseplate wedge protein [*Synechococcus* phage S-H38]	baseplate wedge protein	0	82.8	pfam09215 (2.6E-07)
168	+	121233	128495	2420	VrlC protein [*Synechococcus* phage S-H35]		0	49	
169	+	128495	128737	80	hypothetical protein [*Synechococcus*		2.6E-14	51.4	

				phage S-SCSM1]				
170	+	128727	128924	65	hypothetical protein [*Synechococcus* phage S-H38]		4E-14	66	
171	+	129018	133772	1584	tail fiber protein [*Synechococcus* phage S-H38]	tail fiber	0	60.4	cl33689 (1.6E-10)
172	+	133785	134591	268	neck protein [*Synechococcus* phage S-SSM4]	neck protein	8.9E-130	65.3	PHA02554 (7.4E-88)
173	+	134596	135504	302	neck protein [*Synechococcus* phage S-H38]	neck protein	1.5E-108	67.7	PHA02555 (3.7E-72)
174	+	135504	136307	267	tail sheath stabilizer and completion protein [*Synechococcus* phage S-SSM5]	tail sheath stabilizer and completion protein	8.6E-115	60.7	PHA02556 (1.5E-82)
175	+	136304	136723	139	terminase small subunit [*Synechococcus* phage S-H38]	terminase small subunit (TerS)	1.2E-65	74.1	PHA02585 (2.2E-10)
176	+	136756	137280	174	YadA domain structural protein [*Synechococcus* phage S-P4]		2.7E-51	55.6	
177	+	137282	137611	109	hypothetical protein [*Synechococcus* phage S-H38]		1.2E-52	72.7	
178	+	137615	138889	424	virion structural protein [Cyanophage S-RIM12]		0	69.3	
179	+	138886	139161	91	structural protein [*Synechococcus* phage S-P4]		2.6E-41	81.3	
180	+	139161	139319	52	hypothetical protein [uncultured Mediterranean phage uvMED]		3E-09	55.8	
181	+	139306	140955	549	terminase large subunit [*Synechococcus* phage S-H38]	terminase large subunit (TerL)	0	87	PHA02533 (0)
182	+	141019	143259	746	tail sheath momer [*Synechococcus* phage S-H38]	tail sheath protein	0	63.7	PHA02539 (0)
183	+	143298	144032	244	tail tube momer protein [*Synechococcus* phage S-H38]	tail tube protein	1E-70	54.2	PHA02551 (2.9E-63)

184	+	144081	145727	548	portal vertex protein [*Synechococcus* phage S-H38]	portal vertex protein	0	80.4	PHA02531 (0)
185	+	145755	145922	55	hypothetical protein [*Synechococcus* phage S-H38]		9.7E-09	54.6	
186	+	145922	146569	215	prohead core scaffold protein [*Synechococcus* phage S-H38]	prohead core protein serine protease	3.8E-126	82.3	PHA00911 (4.9E-88)
187	+	146632	147666	344	prohead core scaffold protein [*Synechococcus* phage S-H38]	prohead core protein	4.7E-152	69.9	PHA02557 (3.7E-67)
188	+	147733	149091	452	major capsid protein [*Synechococcus* phage S-H38]	major capsid protein	0	89.9	PHA02541 (0)
189	+	149173	149760	195	tail completion and sheath stabilizer [*Synechococcus* phage S-H38]	tail completion and sheath stabilizer	2.3E-76	56.2	PHA02576 (7.8E-11)
190	+	149788	150216	142	single-stranded DNA binding protein [*Synechococcus* phage S-H38]	recombination, repair and ssDNA binding protein (UvsY)	1E-86	87.3	pfam11056 (3.6E-40)
191	+	150218	151693	491	RNA-DNA + DNA-DNA helicase [*Synechococcus* phage S-H38]	UvsW helicase	0	82.5	PHA02558 (0)
192	+	151686	152120	144	methylamine utilization protein [*Synechococcus* phage S-H38]	RNA-binding protein	1.7E-45	54.9	pfam16243 (1.5E-25)
193	+	152240	152707	155	RNA polymerase sigma factor for late transcription [Cyaphage S-RIM4]	RNA polymerase	3.2E-83	76.6	PHA02547 (2.7E-65)
194	+	152711	153745	344	recombination endonuclease [*Synechococcus* phage S-H38]	recombination endonuclease	0	70.9	PHA02546 (1.5E-150)
195	+	153745	153996	83	hypothetical protein [*Synechococcus* phage S-H38]		1.1E-34	66.3	
196	+	153993	155711	572	recombination endonuclease [*Synechococcus*	recombination	0	64	PHA02562 (0)

					phage S-H38]	endonuclease			
197	+	155929	158070	713	peptidase [*Synechococcus* phage S-H38]		0	52.4	
198	+	158135	159214	359	MoxR family ATPase [*Actimycetia* bacterium]	MoxR family ATPase	0	73.4	cl34016 (1.4E-23)
					cobalamin biosynthesis protein [*Synechococcus* phage S-H38]		6.6E-179	69.6	
199	+	159277	160155	292	S-layer homology domain-containing protein [*Synechococcus* phage S-PM2]	iron uptake porin	2.2E-116	65.5	NF033921 (5.1E-28)
200	+	160213	160521	102	peptidase [*Synechococcus* phage B3]		7E-53	80.8	
201	+	160524	161198	224	sliding clamp DNA polymerase accessory protein [*Synechococcus* phage S-H38]	sliding clamp DNA polymerase accessory protein	3.4E-84	55.4	PHA02545 (1.9E-77)
202	+	161223	162167	314	DNA polymerase clamp loader small subunit [*Synechococcus* phage S-H38]	DNA polymerase clamp loader small subunit	1.7E-176	74.2	PHA02544 (8.3E-159)
203	+	162171	163229	352	putative cytoplasmic protein [*Synechococcus* phage S-SRM01]		0	82.1	
204	+	163226	163609	127	DNA polymerase [*Synechococcus* phage S-H38]	clamp loader small subunit	5.2E-61	72.8	PHA02593 (1.5E-10)
205	+	163606	164031	141	translation repressor protein [bacterium]	translation repressor protein	1.3E-86	89	PHA02543 (9.6E-77)
					endoribonuclease translational repressor of early genes [*Synechococcus* phage S-H38]		1.5E-84	85.7	
207	-	164524	164372	50	hypothetical protein [Cyanobacteria bacterium M_surface_10_m1_298]		3.2E-04	44	
209	+	164906	165358	150	heat shock protein; molecular chaperone IbpA [*Synechococcus* phage S-H38]	heat shock protein (Hsp)	2.4E-68	66.7	PRK10743 (5.3E-32)
210	+	165431	165661	76	hypothetical protein [*Synechococcus*		3.4E-28	60.5	

					phage S-H38]				
211	+	165958	166353	131	hypothetical protein [*Synechococcus* phage S-H38]		1.3E-25	41.6	
212	+	166350	168839	829	DNA polymerase [*Synechococcus* phage S-H38]	DNA polymerase	0	79.1	PHA02528 (0)
213	+	168836	169891	351	recombination protein [*Synechococcus* phage S-H38]	recombination protein	0	89.5	COG0468 (6.3E-24)
214	+	169869	170450	178	2OG-Fe(II) oxygenase [*Synechococcus* phage S-H38]	2OG-Fe(II) oxygenase family protein	1.6E-57	50	smart00702 (3.5E-16)
215	+	170450	171832	460	DNA primase/helicase [*Synechococcus* phage S-H38]	DNA helicase	0	83	PHA02542 (0)
216	+	171841	172299	152	pyrophosphatase [*Synechococcus* phage S-H38]	pyrophosphatase (MazG)	6.7E-68	68.8	cd11541 (8.3E-24)
217	+	172391	173701	436	hypothetical protein [*Synechococcus* phage S-CBM2]		1.4E-162	60.9	
218	+	173735	174868	377	cytitidyltransferase [*Synechococcus* phage S-H38]	nicotinamide/nicotinate mononucleotide adenylyltransferase (NMNAT)	0	71.9	cd02167 (6.3E-06)
219	+	174901	177252	783	hypothetical protein [*Synechococcus* phage S-H38]		8.3E-49	44	
220	+	177263	177910	215	hypothetical protein [*Synechococcus* phage S-H25]		9.2E-34	63.3	

[a]The top and additional homologs for each organism type are displayed based on BLASTP against the non-redundant database.

[b]Putative functions are predicted based on functions of homologs from the Conserved Domain Database.

[c]The best hit of each ORF in the Conserved Domain Database and the corresponding e-value.

Table S2. Predicted ORFs in the S-CREM1 genome with distant homologs detected by using HHpred and Phyre2 search.

ORF	Strand	Left	Right	aa length	Putative function[a]	HHpred search			Phyre2 search		
						Hits	Probability (%)	E-value	Hits	Confidence (%)	Identity (%)
2	+	390	998	202	cell adhesion	no hit			immunoglobulin superfamily; fibronectin; membrane protein, cell adhesion	99.9	21
5	+	2022	2456	144	holin-like protein	holin-like protein [*Bacillus* phage SPP1]	97.8	2.6E-03	no hit		
12	+	8898	9050	50	UvrB	UvrB; DNA excision repair [*Bacillus subtilis*]	96	9.4E-02	no hit		
16	+	11050	11250	66	pre-mRNA-splicing factor	no hit			pre-mRNA-splicing factor 38 [*Chaetomium thermophilum* var. thermophilum DSM 1495]	83.4	25
22	+	14262	17600	1112	collagen-like protein	collagen-like protein [*Acanthamoeba polyphaga* mimivirus]	96.3	2.2E-02	no hit		
28	+	19710	20162	150	DNA ligase	DNA ligase [*Enterobacteria* phage T4]	100	1.3E-28	no hit		
33	+	21773	21919	48	recombination endonuclease VII	recombination endonuclease VII [Bacteriophage T4]	91.3	1.7	no hit		
42	-	27541	26495	348	baseplate tail tube	baseplate-tail tube [*Enterobacteria* phage T4]	100	6.2E-42	baseplate [*Serratia entomophila*]	86	13
44	+	28036	28881	281	tail tube protein	tail tube protein [*Serratia* phage KSP90]	99.9	9.4E-25	tail tube protein [*Enterobacteria* phage T4 sensu lato]	99.9	14

46	+	29669	29842	50	baseplate hub assembly protein	baseplate hub assembly protein [*Enterobacteria* phage T4]	97.1	9.7E-04			
48	+	32965	35940	991	lysozyme murein	no hit			lysozyme murein [*Staphylococcus aureus* subsp. *aureus* Mu50]	92.1	29
49	+	35947	37284	445	tail protein	baseplate central spike complex protein [*Enterobacteria* phage T4]	100	2.7E-35	tail protein [*Neisseria meningitidis* MC58]	98.2	16
54	+	41336	41587	83	YefM-like antitoxin	YefM-like antitoxin [*Escherichia coli*]	92.4	2.3	no hit		
56	+	42086	42304	72	transcription elongation factor	transcription elongation factor SPT5 [*Homo sapiens*]	95.3	7.2E-02	no hit		
63	+	48880	52569	1229	lyase	no hit			lyase [*Bacteroides thetaiotaomicron*]	96	24
67	+	55301	56104	267	tail fiber protein	tail fiber protein [*Acanthamoeba polyphaga* mimivirus]	97	6.3E-02	no hit		
68	+	56135	56623	162	stress response protein	stress response protein [*Bacillus* phage SPbeta]	98	1.7E-05	no hit		
70	+	58331	59068	245	dUTPase	deoxyuridine 5'-triphosphate nucleotidohydrolase (dUTPase) [*Vaccinia* virus]	98	1.9E-03	deoxyuridine 5'-triphosphate nucleotidohydrolase [*Chlorella variabilis*]	94.5	20
73	+	59891	60673	260	2OG-Fe(II) oxygenase family protein	2OG-Fe(II) oxygenase superfamily [Green alga]	96.6	4E-02	no hit		
77	+	62486	63052	188	2OG-Fe(II) oxygenase family	2OG-Fe(II) oxygenase superfamily [*Shewanella baltica*	99.7	1.4E-15	transmembrane prolyl 4-hydroxylase [*Homo sapiens*]	97.9	20

					protein	OS155]					
78	+	63049	63666	205	2OG-Fe(II) oxygenase family protein	2OG-Fe(II) oxygenase superfamily [*Chlamydomonas reinhardtii*]	98.6	2.1E-06	transmembrane prolyl 4-hydroxylase [*Homo sapiens*]	96.2	22
79	+	63663	64256	197	2OG-Fe(II) oxygenase family protein	2OG-Fe(II) oxygenase superfamily [Green alga]	99.8	2.5E-16	prolyl-4 hydroxylase [*Homo sapiens*]	99.8	19
80	+	64253	64780	175	2OG-Fe(II) oxygenase family protein	2OG-Fe(II) oxygenase family protein [*Streptomyces viridochromogenes* Tue57]	99.7	3.2E-15	oxidoreductase activity Fe(II)/(alpha)ketoglutarate-dependent dioxygenase [*Aspergillus stellatus*]	94.3	19
86	+	65930	66115	61	transmembrane protein	transmembrane protein [*Invertebrate iridescent virus 6*]	94.2	1.4	no hit		
98	+	69444	69671	75	high potential iron-sulfur protein	high potential iron-sulfur protein [*Rhodocyclus tenuis*]	93	1.9E-01	no hit		
106	+	71163	71441	103	TacA-like antitoxin	TacA antitoxin peptide [*Salmonella typhimurium*]	98.7	8.4E-08	TacT2 toxin bound to TacA2 antitoxin [*Salmonella enterica* subsp. *enterica* serovar *Typhimurium*]	95.9	32
112	+	72714	73007	97	cadherin	cadherin [*Mus musculus*]	90.3	3.2	no hit		
119	+	75456	75641	61	holin-like protein	holin-like protein [*Bacillus* phage SPP1]	93.17	7.9E-01			
134	+	82293	82499	68	transmembrane protein	transmembrane protein [*Invertebrate iridescent virus*]	95	5.3E-01	no hit		

151	+	88076	88393	105	genomic DNA translation	genomic DNA translation [*Bacillus* phage SP01]	97.6	1.5E-03	no hit		
155	+	89516	89680	54	MazE-like antitoxin	MazE antitoxin [*Escherichia coli*]	99.2	8.2E-10	no hit		
156	+	89733	90080	115	spike glycoprotein	spike glycoprotein [Human coronavirus 229E]	93.4	1.4	no hit		
157	+	90084	94295	1403	lipoprotein lipase	no hit			lipoprotein lipase [*Homo sapiens*]	81.3	30
169	+	128495	128737	80	heat shock protein (Hsp)	heat shock protein [*Methanothermobacter thermautotrophicus*]	94.1	1.7	no hit		
170	+	128727	128924	65	transmembrane signaling receptor activity	transmembrane signaling receptor activity [*Drosophila melanogaster*]	93.9	2.4E-01	signaling protein [*Drosophila melanogaster*]	88	56
176	+	136756	137280	174	ubiquitin-like modifier-activating enzyme	ubiquitin-like modifier-activating enzyme [*Mus musculus*]	94.6	7.9E-01	no hit		
185	+	145755	145922	55	prehead core component	prehead core component [*Enterobacteria* phage T4]	97.2	3.5E-03	no hit		
195	+	153745	153996	83	import protein	import protein [*Plasmodium falciparum* 3D7]	96.7	7.1E-02	no hit		
197	+	155929	158070	713	RNA binding protein	nucleic acid-binding proteins [*Saccharomyces cerevisiae*]	99.4	8.2E-12	RNA binding protein [*Deinococcus radiodurans*]	98.3	20
200	+	160213	160521	102	nucleotide kinase	nucleotide kinase [*Escherichia* phage T7]	99.6	2.5E-15	no hit		

203	+	16217<br>1	16322<br>9	352	iron-binding protein	iron-binding protein that performs methylcarbamoylation of adenine using acetyl CoA [*Escherichia* phage Mu]	99.1	9.4E-10	no hit
210	+	16543<br>1	16566<br>1	76	transcriptional repressor	transcriptional repressor [*Salmonella* bacteriophage P22]	93.9	7.7E-01	no hit

[a]Putative functions are predicted based on functions of homologs identified using HHpred and Phyre2 search.

Table S3. Amino acid sequence identities among the S-CREM1 2OG-Fe(II) oxygenase family proteins.

	ORF75	ORF72	ORF74	ORF136	ORF73	ORF77	ORF78	ORF79	ORF80	ORF214
ORF75	100	24.6	-	30.8	-	-	-	41.2	-	-
ORF72		100	19.8	24.6	-	-	-	-	-	-
ORF74			100	-	-	-	-	-	-	-
ORF136				100	-	-	-	-	-	-
ORF73					100	-	-	-	-	-
ORF77						100	-	34.9	-	-
ORF78							100	-	-	-
ORF79								100	-	-
ORF80									100	-
ORF214										100

Table S4. tRNA genes in the S-CREM1 genome.

tRNA gene	Positions	Strand	Attribute	Anticodon
tRNA1	9057–9129	+	Arg	TCT
tRNA2	9890–9961	+	Met	CAT
tRNA3	10132–10213	+	Leu	TAG
tRNA4	10429–10501	+	Asn	GTT
tRNA5	10572–10643	+	Gly	TCC
tRNA6	10961–11032	+	Ile	TAT
tRNA7	17937–18024	+	Ser	TGA
tRNA8	18025–18098	+	Pro	TGG
tRNA9	37980–38051	+	Val	TAC
tRNA10	41628–41700	+	Leu	TAA
tRNA11	42581–42653	+	Thr	TGT
tRNA12	43886–43958	+	Lys	TTT
tRNA13	43998–44079	+	Tyr	GTA
tRNA14	44084–44155	+	Asp	GTC
tRNA15	44331–44402	+	Glu	TTC
tRNA16	44406–44478	+	Phe	GAA
tRNA17	44576–44648	+	Ile	GAT
tRNA18	44703–44792	+	Ser	GCT
tRNA19	44796–44869	+	Met	CAT
tRNA20	44871–44941	+	His	GTG
tRNA21	44946–45016	+	Gln	TTG
tRNA22	45020–45091	+	Trp	CCA
tRNA23	45095–45168	+	Arg	ACG
tRNA24	155776–155848	+	Ala	TGC

Table S5. Information of cyanophages with tRNA genes more than twenty.

Phage strain	Host	Isolation source	Bona fide tRNA No.	Amino acid specificities	GenBank accession No.	Reference
S-CREM1	*Synechococcus* sp. CB0101	Changjiang River Estuary, surface water	24	19	OP535465	This study
S-CBWM1	*Synechococcus* sp. CBW1002	Baltimore Inner Harbor, surface water	34	20	MG450654	[22]
S-CRM01	*Synechococcus* sp. LC16	Copco Reservoir, Klamath River	33	20	HQ615693	[23]
S-PM2	*Synechococcus* sp. WH_7803	English Channel, 0 m	23	18	AJ630128	[24]

Table S6. Comparison of tRNA complementary codons among cyanophages with tRNA genes more than twenty.

Attribute	Anticodon			
	**S-CREM1**	**S-PM2**	**S-CBWM1**	**S-CRM01**
Arg	ACG	ACG	ACG	ACG
Met	CAT*	CAT**	CAT*	CAT**
Trp	CCA	CCA	CCA	CCA
Ile	GAT	GAT	GAT	GAT*
Ser	GCT	GCT	GCT	GCT
Tyr	GTA	GTA	GTA	GTA
Asp	GTC	GTC	GTC	GTC
His	GTG	GTG	GTG	GTG
Asn	GTT	GTT	GTT	GTT
Leu	TAA	TAA	TAA	TAA
Val	TAC	TAC	TAC	TAC
Gly	TCC	TCC	TCC	TCC
Arg	TCT	TCT	TCT	TCT
Ala	TGC	TGC	TGC	TGC
Pro	TGG	TGG	TGG	TGG*
Thr	TGT	TGT	TGT	TGT
Glu	TTC	TTC	TTC*	TTC
Gln	TTG	TTG	TTG	TTG
Lys	TTT	TTT	TTT	TTT
Leu	TAG	TAG	TAG	-
Phe	GAA	-	GAA	GAA
Ile	TAT	TAT	-	-
Ser	TGA	-	-	-
Ser	-	GGA	-	GGA
Leu	-	-	CAA	CAA
Lys	-	-	CTT	CTT
Val	-	-	GAC	GAC
Leu	-	-	GAG	GAG
Cys	-	-	GCA	GCA
Gly	-	-	GCC	GCC
Thr	-	-	GGT	GGT
Arg	-	-	CCT	-
Thr	-	-	CGT	-
Gln	-	-	CTG	-
Arg	-	-	TCG	-
Glu	-	-	-	CTC

*tRNA genes with specific anticodons found twice in the genome.

**tRNA genes with specific anticodons found thrice in the genome.

Table S7. *wcaG*, *manA*, and *glnA cis*-regulatory RNA genes found in cyanophages.

Feature	Cyanophage strain[a]	Stand	Start	End	Score
wcaG	*Synechococcus* phage S-CREM1	+	9745	9842	91
	Synechococcus phage S-IOM18	+	148008	148102	93.7
	Synechococcus phage S-SM2	+	143437	143532	91.8
	Synechococcus phage S-RSM4	-	29718	29624	89.6
	Synechococcus phage S-WAM2	+	160619	160713	89.6
	Cyanophage S-RIM32	+	169365	169460	89.2
	Synechococcus phage S-SKS1	-	48688	48591	88.9
	Synechococcus phage S-CAM1	-	106335	106239	83.9
	Synechococcus phage S-CAM3	+	186780	186877	82.5
	Synechococcus phage syn9	+	163184	163278	77.9
	Synechococcus phage S-CAM7	+	161007	161102	77.4
	Synechococcus phage S-PM2	+	122806	122905	71.1
	Synechococcus phage S-RIM8 A.HR1	+	157556	157661	62.5
	Cyanophage S-RIM50	+	160772	160877	62.5
	Synechococcus phage ACG-2014e	+	156760	156858	58.8
	Synechococcus phage S-RIM2 R1_1999	+	162743	162837	58
	Synechococcus phage S-CAM22	+	157433	157531	50.1
	Synechococcus phage ACG-2014h	+	155033	155127	49.8
	Prochlorococcus phage Syn1	+	167073	167166	44.2
	Synechococcus phage ACG-2014h	+	155137	155239	43.1
	Prochlorococcus phage Syn1	+	167176	167284	42.4
	Synechococcus phage S-SCSM1	+	107200	107286	37.1
manA	*Synechococcus* phage S-CREM1	+	10215	10429	100.6
	Cyanophage P-RSM6	-	14255	14070	173.2
	Synechococcus phage metaG-MbCM1	+	113853	114040	173.1
	Prochlorococcus phage P-SSM2	+	142837	143034	171.3
	Prochlorococcus phage P-HM1	+	112740	112927	170.7
	Prochlorococcus phage P-SSM4	+	164829	165014	168.7
	Prochlorococcus phage P-TIM68	+	152799	152994	166.3
	Prochlorococcus phage P-SSM7	+	171456	171649	165.4
	Prochlorococcus phage P-RSM4	+	120783	120974	161.2
	Prochlorococcus phage P-HM2	+	4771	4969	160.4
	Synechococcus phage S-SSM5	+	116393	116586	160.1
	Cyanophage P-TIM40	+	169071	169265	136.9
	Cyanophage P-RSM1	-	121342	121143	126.1
	Cyanophage S-SSM4	+	130075	130274	122.9
	Synechococcus phage S-SSM7	+	151363	151563	122.8
	Synechococcus phage ACG-2014f	+	128566	128776	91.4
glnA	*Synechococcus* phage S-CREM1	-	164330	164232	44
	Prochlorococcus phage P-TIM68	+	193968	194062	46.7
	Synechococcus phage ACG-2014f	+	213517	213608	44.9
	Synechococcus phage S-PM2	+	142315	142415	43.3

Synechococcus phage S-ShM2	+	162519	162753	42.7

[a]*cis*-regulatory RNA genes in the S-CREM1 genome were predicted by searching against the Rfam database. Information of *cis*-regulatory RNA genes of other cyanophages were obtained from the Rfam database.

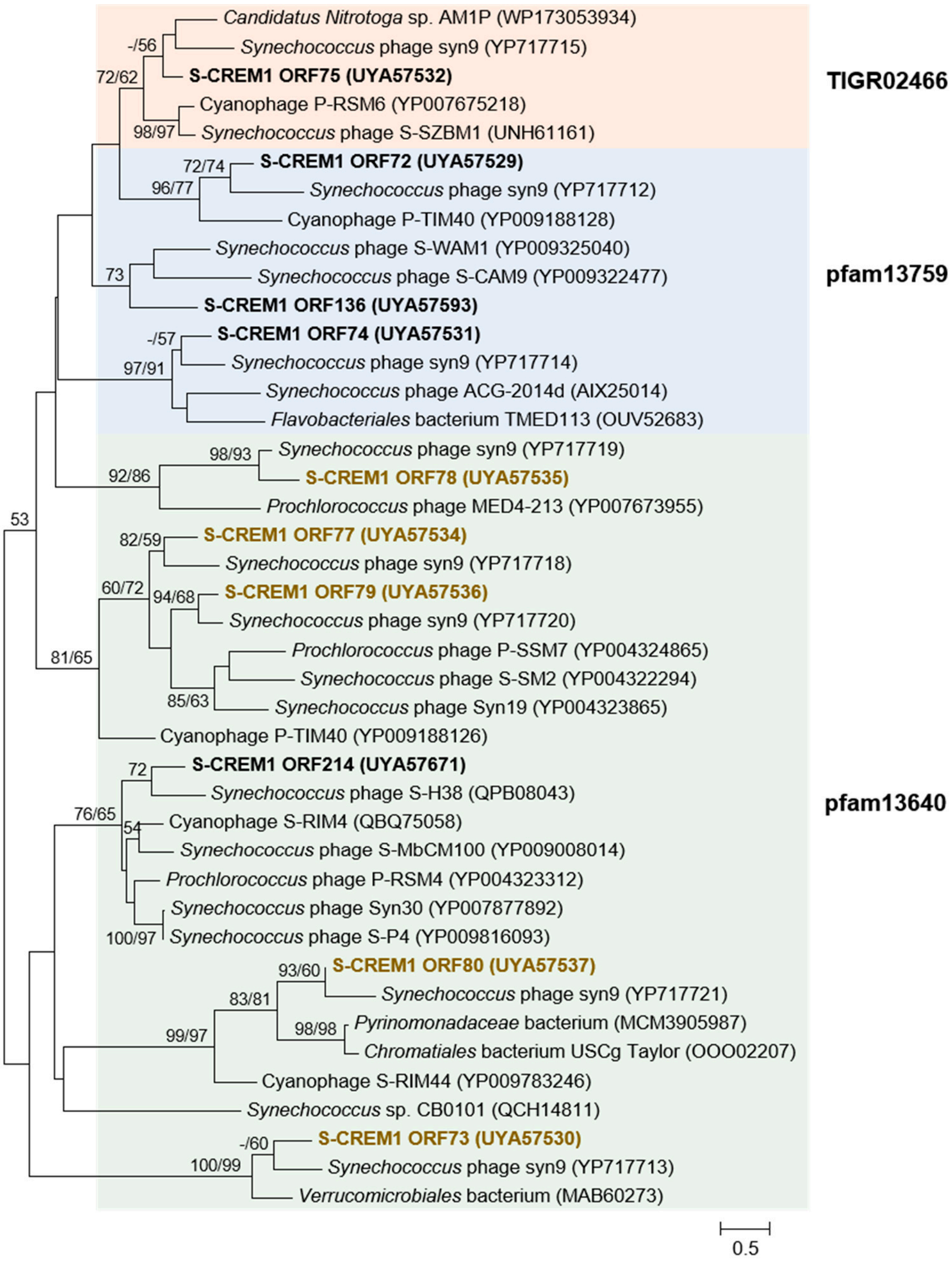

Figure S1. Unrooted maximum likelihood phylogenetic tree of the S-CREM1 predicted 2OG-Fe(II) oxygenase superfamily proteins. ORFs in brown font were identified based on predicted structural properties using HHpred and Phyre analyses. Bootstrap values (maximum-likelihood/neighbor-joining) are based on 1,000 replicates, and only values > 50% are shown.

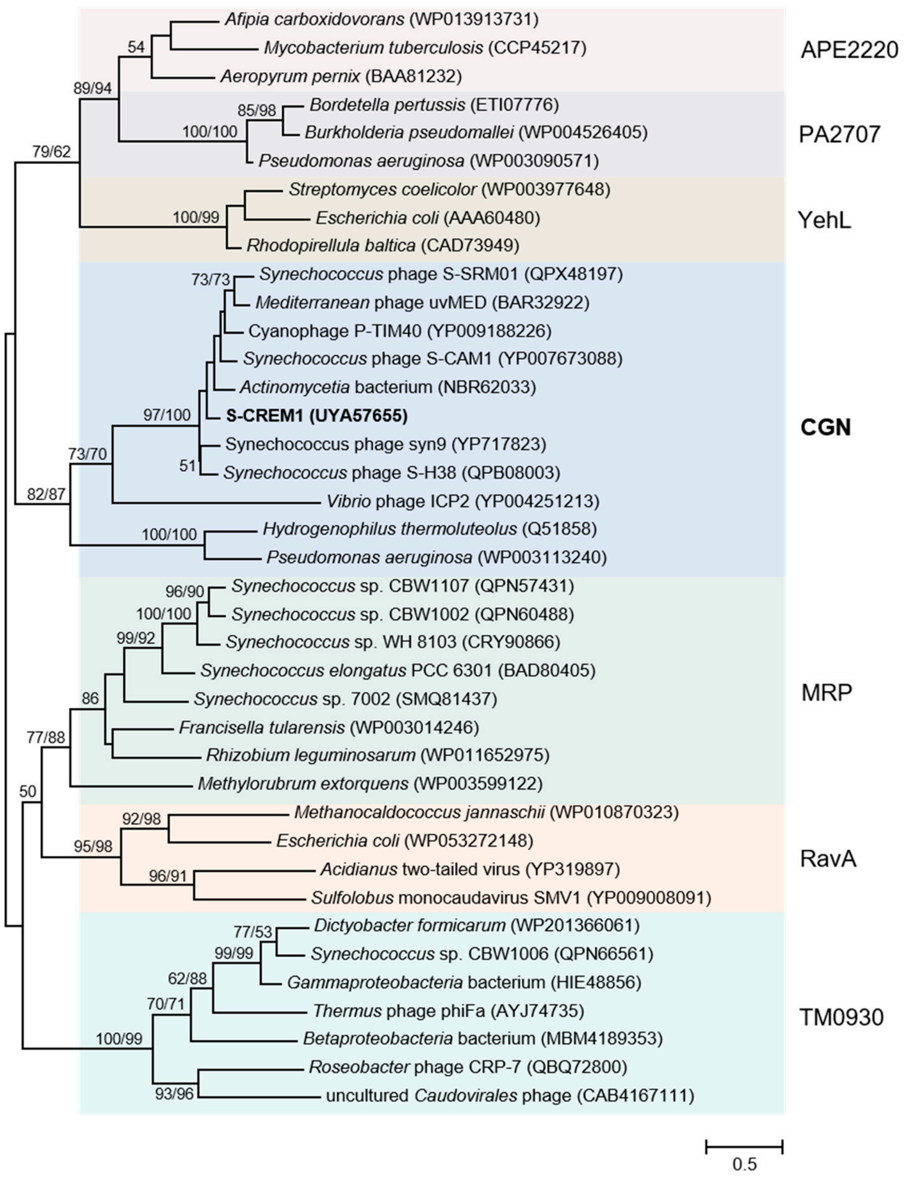

Figure S2. Unrooted maximum likelihood phylogenetic tree of the MoxR ATPases. The number of bootstrap replicates = 1,000. The bootstrap values (maximum-likelihood/neighbor-joining) are shown near each node, and only values > 50% are shown.

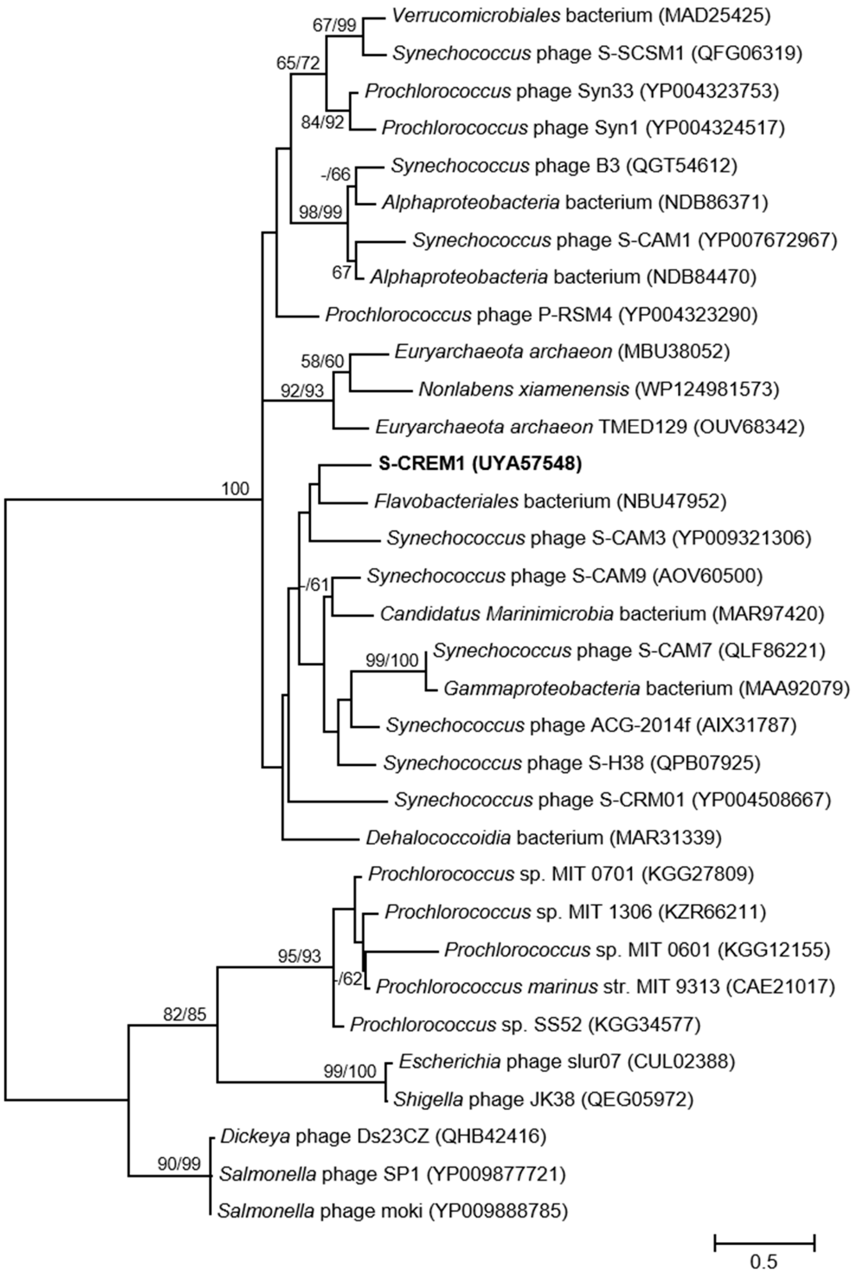

Figure S3. Unrooted maximum-likelihood phylogenetic tree of the S-CREM1 pyrimidine dimer DNA glycosylase. The bootstrap values (maximum-likelihood/neighbor-joining) of > 50% are shown near each node. The number of bootstrap replicates = 1,000.

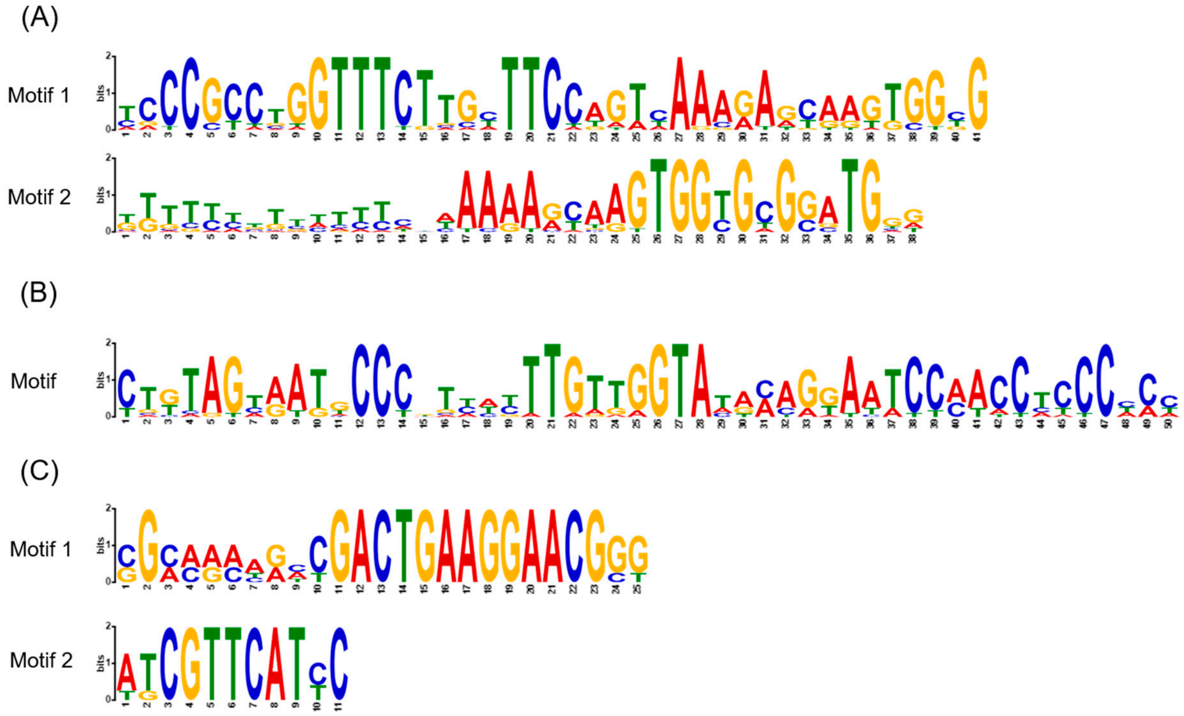

Figure S4. DNA sequence conserved motifs in *wcaG* (A), *manA* (B), and *glnA* (C) *cis*-regulatory RNA genes among S-CREM1 and other cyanophages in the Rfam database.
